# First Evidence of Metabolically Active Intracellular Bacteria in *Saccharomyces cerevisiae*

**DOI:** 10.1021/acs.jafc.5c04823

**Published:** 2025-10-07

**Authors:** Annabella Tramice, Gianni Liti, Annalaura Iodice, Gennaro Roberto Abbamondi, Federica Carlea, Ernesto Petruzziello, Adele Cutignano, Debora Paris, Carmine Iodice, Matteo De Chiara, Maria Aponte, Francesca De Filippis, Chiara Vischioni, Andrea Motta, Giuseppe Blaiotta, Giuseppina Tommonaro

**Affiliations:** † 201790 Institute of Biomolecular Chemistry, Consiglio Nazionale delle Ricerche, 80078 Pozzuoli, NA, Italy; ‡ Université Côte d’Azur, CNRS, INSERM, IRCAN, 06107 Nice Cedex 2, France; § Department of Agricultural Sciences, Federico II University of Naples, 80055 Portici, NA, Italy; ∥ Task Force on Microbiome Studies, Federico II University of Naples, 80126 Naples, Italy

**Keywords:** *Saccharomyces cerevisiae*, tyrosol, N-acylhomoserine lactones, quorum sensing, metataxonomic analysis

## Abstract

Quorum sensing (QS) is a cell-to-cell signaling system that takes place at a key concentration (quorum) of signal molecules and via a peculiar signaling pathway. Both bacteria and yeasts possess QS mechanisms, mediated by specific molecules (farnesol, tyrosol, 2-phenylethanol, tryptophol) in yeasts, and *N*-acylhomoserine lactones (AHLs) and modified oligopeptides in bacteria. Here, we report the first chemical evidence of bacterial QS activity in yeast *Saccharomyces cerevisiae* (OS3 and V5 strains) by UPLC-MS/MS identification of *N*-octanoyl- and *N*-decanoyl-L-homoserine lactones in cell-free culture media extracts. The AHLs' presence was unexpected, as they are produced exclusively by bacteria. Tyrosol, a yeast signal molecule, was identified and quantified by NMR analysis. Metataxonomic analysis suggested the existence inside *S. cerevisiae* cells of bacteria, including *Firmicutes*, *Bacteroidota*, and *Proteobacteria*. Our study paves the way for investigations into bacterial detection within *S. cerevisiae* cells and their role in biotechnological performance in the food fermentation fields.

## Graphical abstract

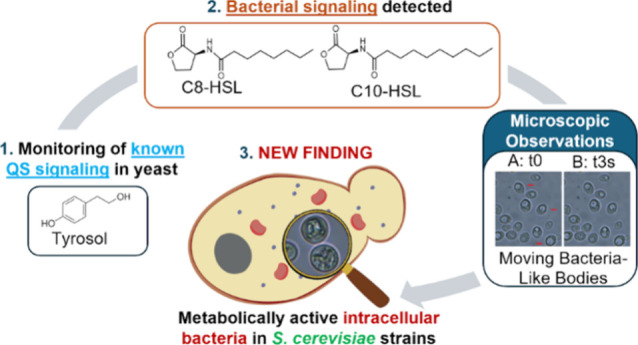

## 1 Introduction

Yeasts and bacteria can coexist in the same habitat. Several studies have investigated their interactions, and the existence of yeast endosymbiotic bacteria has been described in a few cases.^1^ *Bacillus tequilensis* was reported to establish an endosymbiontic relationship with a peculiar strain of *Kluyveromyces marxianus*, which was isolated from *Agave durangensis* fermentation processes. *B. tequilensis* easily uses the intracellular microaerobic cell environment and protects its nitrogenase complex from oxidative damage.^2^ The pathogenic *Helicobacter pylori* enters eukaryotic cells of humans or other species, including yeasts of the genus *Candida*, when subjected to stress conditions (such as pH changes or scarce nutrients): *Candida* cells harbor this bacterium and become transmission vehicles for it.^3^ *Staphylococcus hominis* and *Staphylococcus hemolyticus* were localized inside the vacuole of two *C. albicans* yeasts, and for the first time, the release and cultivability of these intracellular bacterial cells from yeast were described.^4^ Inside the yeast cytoplasm of *C. tropicalis* was thought to catabolize starch due to the presence of *Microbacterium* sp. growing in its cytoplasm and providing a stable habitat for *Microbacterium* sp.^5^ All the above reports demonstrated the endosymbiotic relationship by localizing the guest bacteria inside the yeast host cells, and eventually defining the benefit that this coexistence could provide each other; however, to date, the occurrence of a molecular signaling regulating yeast and endobacteria communities involved in the consortia has been not disclosed. More precisely, two questions are unanswered: (i) do yeast and bacteria use a chemical language to communicate? and (ii) would this knowledge be relevant in biotechnological applications?

In this paper, our attention was focused on *Saccharomyces cerevisiae* yeast*:* it is a budding yeast with a recognized Generally Regarded as Safe (GRAS) status by the FDA.^6^ It is the best-studied eukaryotic experimental model organism, and it is used in industrial fermentations for producing a broad range of fermented foods (bakery and dairy products), beverages (wine, beer, and cider), biofuel, and pharmaceutical products.^7,8^ During grape maceration for wine production, several interactions take place between *S. cerevisiae* and other microorganisms (e.g., non-*Saccharomyces* yeast, filamentous fungi, lactic acid, and acetic acid bacteria) involved in the fermentation process. These interactions are mainly due to the production of small molecules, namely quorum-sensing molecules (QSMs), which act on cellular density and could affect the quality of the final product.^9^ It is known that QS is a specific form of intercellular signaling (IS) that can facilitate communication, chemical cues, or chemical manipulation between microorganisms. It involves a cell-density-dependent regulation of gene expression triggered by the attainment of a critical concentration of small diffusible molecules, named autoinducers (AIs).^10,11^ In bacteria, different AIs are reported: (i) *N*-acylhomoserine lactones (AHLs), mainly produced by Gram-negative; (ii) modified peptides, mainly produced by Gram-positive; and (iii) autoinducer-2 (AI-2), produced by bothGram-positive and Gram-negative bacteria, which acts as a potentially “universal” signal for regulating intraspecies and interspecies communication.^12–14^

The existence of QS systems in fungi has been recently discovered in *C. albicans*: farnesol and tyrosol were identified as QSMs, and both are able to regulate growth, morphogenesis, biofilm production, cell adhesion, and motility.^15,16^ Other fungal QSMs include 2-phenylethanol and tryptophol.^17,18^ In *S. cerevisiae*, the QS usually controls morphogenesis. In fact, *S. cerevisiae* can exist in different multicellular forms depending on its environment, which include sessile and planktonic cells, colonies, biofilms, filaments, mats, flocs, and floes.^19^ Recent studies have suggested that *S. cerevisiae* is able to secrete the aromatic alcohols 2-phenylethanol, tryptophol, and tyrosol as QSMs that regulate control morphogenetic changes in nitrogen starvation conditions.^9,17,20,21^ Together with their relevant biological role, 2-phenylethanol, tryptophol, and tyrosol show important biotechnological applications, especially in wine quality assessment, aroma production in food and drinks, and they can also act as antioxidants, antimicrobials, and/or disinfectants.^22,23^ In the search for new selected wine yeasts, *Hanseniaspora uvarum*, *Torulaspora pretoriensis*, *Zygosaccharomyces bailii*, and the commercially used starter culture *S. cerevisiae* Lalvin EC1118 were also investigated for the production of 2-phenylethanol, tryptophol, and tyrosol as QSMs in peculiar fermentation conditions.^21^

Here we present for the first time the chemical evidence of the possible existence in *S. cerevisiae* (OS3 and V5 strains) of bacteria localized into their yeast cells: typical bacterial QSM such as *N*-octanoyl-l-homoserine lactone (C8-HSL) and *N*-decanoyl-l-homoserine lactone (C10-HSL) were unexpectedly detected by highly sensitive TLC-overlay assay and subsequently identified unequivocally by UPLC-MS from the cell-free extract of *S. cerevisiae* OS3 and V5 strains. The typical yeast QSM tyrosol was also identified by NMR analysis. Further suggestions on the presence of bacteria inside the cells of yeast were given by metagenomic analyses, DNA staining, fluorescent microscopy, and a preliminary enzymatic screening on OS3 and V5 yeast cellular systems.

## 2 Materials and Methods

### 2.1 Chemicals and Reagents

AHL standards (C8-HSL, 3-oxo-C6-HSL, and 3-oxo-C10-HSL), mono- and polysaccharides, ethyl acetate, acetonitrile, methanol, acetic acid, 2-propanol, formic acid, 5-bromo-4-chloro-3-indolyl-β-D-galactopyranoside (X-gal), gentamycin solution (30 μg mL^–1^), chloramphenicol, and cycloheximide were purchased from Merk Life Science S.r.l (Milan, Italy). Reverse-phase silica gel and TLC silica gel plates were purchased from VWR International (Milan, Italy). Water for LC-MS analysis was obtained by a Milli-Q apparatus (Millipore, Milan, Italy).

### 2.2 Yeast Strains

*Saccharomyces cerevisiae* OS3 (DBVPG 6765; NCYC 3264)^24^ and *S. cerevisiae* V5 (M6–3)^25^ were the strains used in this study.

Strains were stored in Malt Extract Broth (Oxoid) containing 20% glycerol (Sigma) at −20 °C. Routine cultures were maintained in WL Nutrient Agar (Oxoid) slants at 4–6 °C. Working cultures were grown in YPD (10 g/L yeast extract, 20 g/L peptone, 20 g/L dextrose). Purity was tested by streaking on WL Nutrient Agar and simultaneously observing under a microscope.

### 2.3 Growth of *S. cerevisiae* OS3 and V5 in Chemically Defined Medium

Growth was monitored in 0.67% Yeast Nitrogen Base without amino acids (YNBwAA) (Sigma, Y0626) containing 20 g/L dextrose in triplicate. Water and dextrose solutions were sterilized by autoclave treatment; 10 × YNBwAA solutions were sterilized by filtration. Sterile solutions were mixed in sterile conditions to obtain the final medium (3 × 2.8 L total volume in a 5.0 L sterile Duram bottle containing a stirring bar). The strain OS3 was pre-grown in YPD broth, containing 100 mg/L chloramphenicol, at 30 °C until the OD600 nm (Honda spectrophotometer V10-Plus) reached 1.6–1.8, and then was inoculated in YNBwAA at a rate of 1%. The growth was monitored for 72 h at 30 °C under static conditions. Uninoculated medium (CN), inoculated medium at the beginning of fermentation (t_0_ h), and at different times during the growth (*t* = 6, 11, 24, 48, and 72 h) were sampled. Before each sampling, the cultures were stirred at 200 rpm for 5 min. The growth was monitored by evaluating the OD and by total viable yeast counts on WL Nutrient Agar containing 100 mg/L chloramphenicol at 30 °C for 5 days. Bacterial contamination was evaluated by plating 0.1 mL on PCA (Oxoid) and on MRS agar (Oxoid) containing 40 mg/L of cycloheximide to inhibit *Saccharomyces*, and by simultaneously observing under a microscope (Nikon Eclipse E400). During fermentation, the pH was monitored by using a pH 60 VioLab (XS instruments), while residual glucose and produced ethanol, succinic and acetic acids, and glycerol were determined by high-performance liquid chromatography (HPLC) analyses as previously described.^25^ Samples for chemical analyses (200 mL each) were collected at *t* = 0, 1, 2, 3, 4, 5, 6, 12, 24, 48, and 72 h and immediately centrifuged 6000 rpm (4800×*g*) for 10 min (Rotor A8–50, Centrifuge NEYA8) to separate cells from fermented broth, and both were stored at −80 °C.

*S. cerevisiae* V5 strain was cultured as described for the OS3 strain (see above), but samples for chemical analyses were collected at the key time points (t = 0, 6, and 24h) for the determination of tyrosol content, the detection and identification of AHLs, and enzymatic activities.

### 2.4 Microscope Observation

Two different approaches were used for the microscope observations. Yeast cultures, stored at −20 °C, were grown in YPD medium containing 100 mg/L chloramphenicol and then inoculated on different media: WL Nutrient Agar (WLNA, Oxoid) supplemented with 40 mg/L of cycloheximide; Nutrient Agar (NA, Oxoid) supplemented with 40 mg/L of cycloheximide; Baird Parker Agar Base supplemented with Egg’s Yolk Tellurite Steril Emulsion (BP-EYT) (Schalau); *Pseudomonas* agar Base with CFC Selective supplement (PA-CFC) (Schalau); Yeast Pre-Sporulation Medium (YPSM: yeast extract 10 g/L; dextrose, 100 g/L; potassium acetate, 20 g/L; agar, 20 g/L); Yeast Sporulation Medium (YSM: yeast extract 10 g/L; dextrose, 0.1 g/L; potassium acetate, 10 g/L; agar, 20 g/L). All cultures were incubated at 30 °C for 5–10 days. All grown cultures were analyzed by light microscopy, and images and video were captured by VisiCam 10.0 (VWR International).

Six μL of the cell suspension was also placed on a microscope slide, coverslips were placed on the slides, and the samples were observed under an optical microscope (Axio Imager D2—Carl Zeiss Microscopy, Jena, Germany) fitted with a 63× and 100× objective lens and an attached camera (Axiocam MRm—Carl Zeiss Microscopy, Jena, Germany). Fiji was used to generate the scale bar. Moreover, with the attempt to verify the contamination by anaerobic bacteria, yeast colonies grown on BP and YSM, in which Moving Bacteria-Like bodies (MBLBs) were present, were inoculated in (a) YCFAGSC medium^26^ supplemented with 40 mg/L of cycloheximide; (b) BBA-HVK, Brucella Blood Agar with Hemin and Vitamin K1 (Becton Dickinson GmbH) with 40 mg/L of cycloheximide; (c) CMM, Chopped Meat Medium (DSMZ no. 78)^27^ supplemented with 40 mg/L of cycloheximide. All media were incubated anaerobically at 37 °C.

### 2.5 Starvation Protocol

The used protocol was as described by Yue et al.^24^ Briefly, cells were first pulled out from glycerol stocks in solid YPD (23 °C, overnight); subsequently, they were streaked to confirm that there was no bacterial contamination. A single colony was cultivated in liquid YPD (2% dextrose, 1% yeast extract, 2% peptone) for 24 h, at 23 °C. Afterward, cells were diluted 1:50 in 10 mL of presporulation medium (YPA: 2% potassium acetate, 1% yeast extract, 2% peptone) and incubated for 48 h at 23 °C (shaking 220 rpm). Presporulated cells were finally resuspended in 25 mL of Sporulation medium (2% KAc) in 250 mL flasks. The flasks were kept at 23 and 30 °C and shaken at 220 rpm for 96 h. The samples were observed under an optical microscope in each of the three steps of the protocol.

### 2.6 MBLBs Visualization by DNA Dyes

To localize the MBLBs inside the yeast cells, a single colony of each strain (OS3 and V5), was inoculated in 10 mL of YPD. The tubes were incubated for up to 6 days at 23 °C under shaking conditions (220 rpm). The samples were stained with the LIVE/DEAD BacLight Bacterial Viability Kit (Cat. No. L7012) according to the manufacturer’s instructions. Briefly, the cells were washed with 0.85% NaCl and supplemented with 3 μL of the dye mixture for each milliliter of the yeast suspension. The cell suspension was mixed thoroughly and incubated in the dark for 15 min before the microscopy analysis. A fluorescent microscope (Axio Imager D2) fitted with a 63× and 100× objective lens and an attached camera (Axiocam MRm) was used for visualization and image capture. Photographs were taken at different time intervals to reveal the movement of the BLBs. Fiji was used to process the images.

### 2.7 Extraction of Cell-Free Medium

The spent medium (200 mL) from *S. cerevisiae* OS3 and V5 strains cultures at different growth stages (*t* = 0, 6, 12, 24, 48, and 72 h) was centrifuged as previously reported.^28^ The supernatants were then extracted with ethyl acetate (1:1 v/v; three times) and subsequently dried under vacuum at *T* < 40 °C. For each growth time, three media extracts were collected and investigated by MS spectrometry and NMR spectroscopy. The extracts were dissolved in methanol to a final concentration of 0.6 mg/mL and directly analyzed by UPLC-MS/MS. Additionally, the extracts were tested for the detection of QS signal molecules by means of the TLC-overlay assay.

### 2.8 QSMs Identification and Characterization

#### 2.8.1 Identification of Fungal QSMs by NMR Analysis

One-dimensional (^1^HNMR) and two-dimensional homonuclear and heteronuclear (TOCSY, HSQC, HMBC-NMR) spectra were acquired on a Bruker AVANCE III spectrometer operating at 600 MHz for proton, equipped with a CryoProbe and an automatic and cooled sample changer. For acquisition, 5–25 mg of ethyl acetate extracts of cell-free spent medium were dissolved in 0.700 mL of MeOD containing 0.03% v/v of TMS (Tetramethylsilane, 136.08 μg for each experiment used as internal standard), and the solutions were placed in a 5 mm NMR tube.

Standard solutions of tyrosol, farnesol, 2-phenylethanol, and 2-phenoxyethanol were prepared (5 mg of sample in 0.700 mL of MeOD with 0.03% v/v of TMS) and compared to the ethyl acetate extract spectra.

Set to 1 the value of the integral of the 12 protons of TMS at 0 ppm, ^1^H integrals of two aromatic tyrosol signals at 7.02 ppm were measured at different times of broth growth, and a quantification of produced tyrosol was made according to the following formula:

$$\mu g\;\mathrm{tyrosol}/\mathrm{mL}=[(A_{7.02\mathrm{ppm}}\times \mu g_{\mathrm{TMSsta}}/A_{\mathrm{TMS}-\mathrm{sta}})\times 2/12]/V_{\mathrm{sample}}$$

where:

*A*_7.02 ppm_ = Area of signal corresponding to 2 aromatic protons of tyrosol at 7.02 ppm;

*A*_TMS‑sta_ = Area of signal corresponding to 12 protons of TMS at 0 ppm, which was calibrated to 1;

μg_TMSsta_ = 136.08 μg of TMS used in each ^1^H NMR experiment;

2/12 = Ratio of the number of protons responsible for signals at 7.02 ppm of tyrosol and 0 ppm of TMS;

*V*_sample_ = 200 mL, the total volume of spent medium collected at different times.

#### 2.8.2 Identification of Bacterial QSMs

##### 2.8.2.1 TLC-Overlay Assay

Ethyl acetate extracts (2 mg) of the cell-free spent medium and standards (2 μL 3-oxo-C6-HSL 10 μM and 2 μL 3-oxo-C10-HSL 400 μM) were applied to RP-C_18_ thin-layer chromatography (TLC) plates (20 × 20 cm; VWR International) and developed by using 60% (v/v) aqueous methanol as mobile phase. The TLC plates were overlaid with 100 mL of ATGN^29^ soft agar (0.6% w/v) supplemented with 0.5% glucose, 40 μg mL^–1^ X-Gal (5-bromo-4-chloro-3-indolyl-beta-D-galactopyranoside), antibiotic (gentamycin, 30 μg mL^–1^), and inoculated with the bioreporter *Agrobacterium tumefaciens* NTL4 (pZLR4) (overnight culture), able to detect AHLs with medium-chain length.^30^ The TLC plates were kept in a sterile container and incubated at 30 °C for 24–48 h.

##### 2.8.2.2 UPLC-MS/MS Analysis

UPLC-MS/MS analyses were performed as reported in Abbamondi et al.,^31^ with a slight modification. Briefly, chromatographic runs were acquired on an Acquity UPLC System (Waters, Milford, MA, USA) coupled to a 3200 API Triple Quadrupole mass spectrometer (Sciex, Foster City, CA, USA) with a Turbo VTM interface equipped with a turbo ion spray probe used in positive ion mode and on an Acquity UPLC BEH C18 column (100 × 2.1 mm, i.d. 1.7 μm, Waters, Milford, MA, USA). A water/ACN (9:1, v/v) mixture was used as eluent A and ACN (100%) as eluent B. A linear gradient profile was programmed from 100% A to 100% B in 1.0 min and remained constant over 3.0 min, followed by a re-equilibration step of 5 min. Separations were performed at a temperature of 60 °C, using a flow rate of 0.7 mL min^–1^ and an injection volume of 5 μL.

Multiple Reaction Monitoring (MRM) experiment was used to collect data by setting the following source parameters: curtain gas (N_2_), 20 psi; ion source gas (GS1), 55 psi; turbo-gas (GS2), 70 psi; desolvation temperature, 550 °C; collision activated dissociation gas (CAD), 4 au; and ion spray voltage, 5500 V. The ions monitored in Q1 included the parent AHL [M + H]^+^, while in Q3, the lactone moiety at *m*/*z* 102 was monitored. Analyst software (version 1.6.2; SCIEX) was used for data acquisition and analysis.

A quantitative method was developed by external standard calibration based on nine calibration points (3, 5, 10, 15, 30, 50, 100, 150, and 300 ng/mL) of the C8-AHL and C10-AHL standards, in triplicate. The response was linear in the selected range with *R* ^2^ > 0.992 in both cases.

For sample analysis, ethyl acetate extracts (9–18 mg) of the cell-free spent medium (200 mL) collected at the growth times of 12, 24, 48, and 72 h (previously analyzed by TLC-overlay assay) in triplicate were dissolved in 500 μL of methanol. The amount of QSMs in the extract was obtained by interpolation of the calibration curve and expressed as the nanomolar concentration in the culture medium.

### 2.9 Enzymatic Investigation

Cell pellets of OS3 and V5 strains isolated from the culture medium whole were suspended in PBS (Phosphate Buffered Saline) (1×, pH 7.4), with a ratio of 1:7 (mg of humid cells/μL of used buffer) and disrupted as previously described by Tramice et al.^28^ Cells were disrupted by sonication for 3 min and homogenized by ULTRA-TURRAX for a further 2 min in an ice–water bath. Cell debris was removed by centrifugation at 5524×g at 4 °C for 45 min.

The cell-free supernatants from different growth conditions of *S. cerevisiae* OS3 and V5 strains were tested for their protein content (Table S1), stored at −80 °C, and used as crude enzyme preparations in enzymatic digestions. Protein concentration was routinely estimated using the Bio-Rad Protein System, and bovine serum albumin was used as standard.^32^

The crude protein enzymatic extracts were tested for the presence of α- and β-glucanase activities by using several substrates such as starch, amylose, amylopectin, pullulan, glycogen, β-glucan from barley, curdlan, laminarin, laminaribiose, and laminaripentaose. Solutions of 5 mg/mL of each substrate in 100 mM sodium acetate buffer at pH 5 were incubated at 40 °C under magnetic stirring in the presence of a fixed protein amount of the four extracellular enzymatic solutions obtained from different growth conditions.

All reactions were carried out using 60 μg of total protein per milligram of reagent. Reactions were monitored over time (0–48 h) by TLC analysis. The TLC solvent system used was EtOAc:AcOH:2-Propanol:HCOOH:H_2_O, 25:10:5:1:15 by vol. Compounds on TLC plates were visualized under UV light or charring with α-naphthol reagent.

The enzymatic digestions of pullulan, amylopectin, and laminarin were stopped in an ice-bath at 48 h for 5 min, and 0.100 mL of each sample was assayed for the glucose equivalent production (DNS assay).^33^ The tests were conducted in triplicate, and the calibration curve of glucose was elaborated in a concentration interval of 0.05:1 mg mL^–1^. Moreover, fasta36 was used to search for pullulanase and laminarase sequences in the strains data set at EVOMICS,^34^ and in the pangenome.^35^

### 2.10 DNA Isolation and Meta-Taxonomic Analysis

The yeast culture showing MBLBs was grown on YSM and BP-YET for 5 days. Cells were harvested from agar plates and washed with sterile water, and DNA was isolated using a Nucleo Spin Food kit (Macherey-Nagel) following the supplier's instructions. Bacterial communities were assessed by HTS of the amplified V3–V4 regions within the 16S rRNA gene (∼460 bp). PCR was carried out with primers (S-D-Bact-0341-*b*-S-17/S-D-Bact0785-a-A-21) connecting with barcodes as previously described by Aponte et al.^36^ PCR products with proper sizes were selected by 2% agarose gel electrophoresis. The same amount of PCR products from each sample was pooled, end-repaired, A-tailed, and further ligated with Illumina adapters. The library was checked with Qubit and real-time PCR for quantification and a bioanalyzer for size distribution detection. Quantified libraries were pooled and sequenced on a paired-end Illumina platform to generate 250 bp paired-end raw reads. Paired-end reads were joined using FLASH.^37^ The DADA2 method^38^ was used for noise reduction. ASVs (Amplicon Sequence Variants) were further filtered using QIIME2 software (Version QIIME2–202202) and identified using the Silva Database 138.1.

### 2.11 Alignment of *Lux* Genes against *S. cerevisiae* Assemblies

To exclude the presence of Lux genes as HGT integrated inside yeast genomes, we put together all the long-reads assemblies two studies^24,39^ among which is also available OS3 (as DBVPG6765), and we used both Blastn and Tblastx (BLAST suite v. 2.13.0) to search for the presence of LuxR (sequence searched: NC_011186.1:1162619-1163371) and acyl-homoserine-lactone synthase (sequences searched: NZ_SILH01000001.1:2962926-2963591, NZ_MRDH01000005.1:140513-141178, NZ_CP098803.1:3577150-3577815, NZ_JAAXQT010000001.1:c446238-445573, NZ_CP071612.1:3025255-3025920, NZ_CP071454.1:424262-424927, NZ_FYAH01000010.1:25839-26489, NZ_CP031495.1:286275-287471, NZ_CP009467.1:2502877-2504076, NZ_AP025466.1:c1546624-1545446). Reads for DBVPG6765 ^24^ were also mapped with the Burrow-Wheeler Aligner (BWA) software against a multi-FASTA, containing *Saccharomyces* representatives and the previously mentioned LuxR and acyl-homoserine-lactone synthase.

### 2.12 Statistical Analysis

A One-way repeated measures ANOVA test was conducted to compare the amount of tyrosol, C8-AHL, and C10-AHL expressed in *S. cerevisiae* samples collected at different time points during the fermentation process. Once the normal distribution of the data set, Mauchly’s test of sphericity was applied to the data, and Greenhouse-Geisser (ε < 0.75) or Huynh-Feldt (ε near or above 0.75) corrections were used for the within-subjects effect in case of sphericity violation. Multiple comparisons were assessed with the Bonferroni correction method (Table S2). Statistical tests were elaborated with the OriginPro 9.1 software package (OriginLab Corporation, Northampton, USA) and R software.^40^

## 3 Results

### 3.1 Growth Monitoring of *S. cerevisiae* OS3 and V5 in Chemically Defined Medium

*S. cerevisiae* OS3 (DBVPG 6765; NCYC 3264) strain was originally isolated from lychee fruit (Indonesia).^24^ The monitoring of its growth in Yeast Nitrogen Base without amino acids (YNBwAA) medium was performed. Figure 1 shows the growth kinetics of the OS3 strain and the production of the main metabolites during 72 h of fermentation at 30 °C. In the first 12 h, the strain reached the stationary phase. In fact, viable counts rapidly increased from 5.71 ± 0.15 log CFU/mL at 0 h to 7.44 ± 0.24 log CFU/mL after 12 h. The last level was maintained constantly until 48 h to slightly decrease at 72 h, reaching 6.97 ± 0.15 log CFU/mL (Figure 1A). An opposite behavior was observed for the pH: a rapid decrease in the first 12 h (from 5.13 to 2.86 units) was recorded with a stationary value for the rest of the monitoring time (Figure 1A). The glucose of the medium was depleted in the first 24 h, and consequently, ethanol reached its maximum level (about 8.0 g/L) (Figure 1B). In this phase, glycerol and succinic acid also reached their maximum production levels, i.e., =1.3 and 0.6 g/L, respectively. The strain V5, isolated from Passito wine (Italy),^25^ monitored for 24 h in the same medium, showed similar growth performances reaching an optical density of 1.764 OD600, a viable count of 7.35 ± 0.05 log CFU/mL, and a pH of 2.83 (data not shown) after 24 h of fermentation.

**Figure 1:**
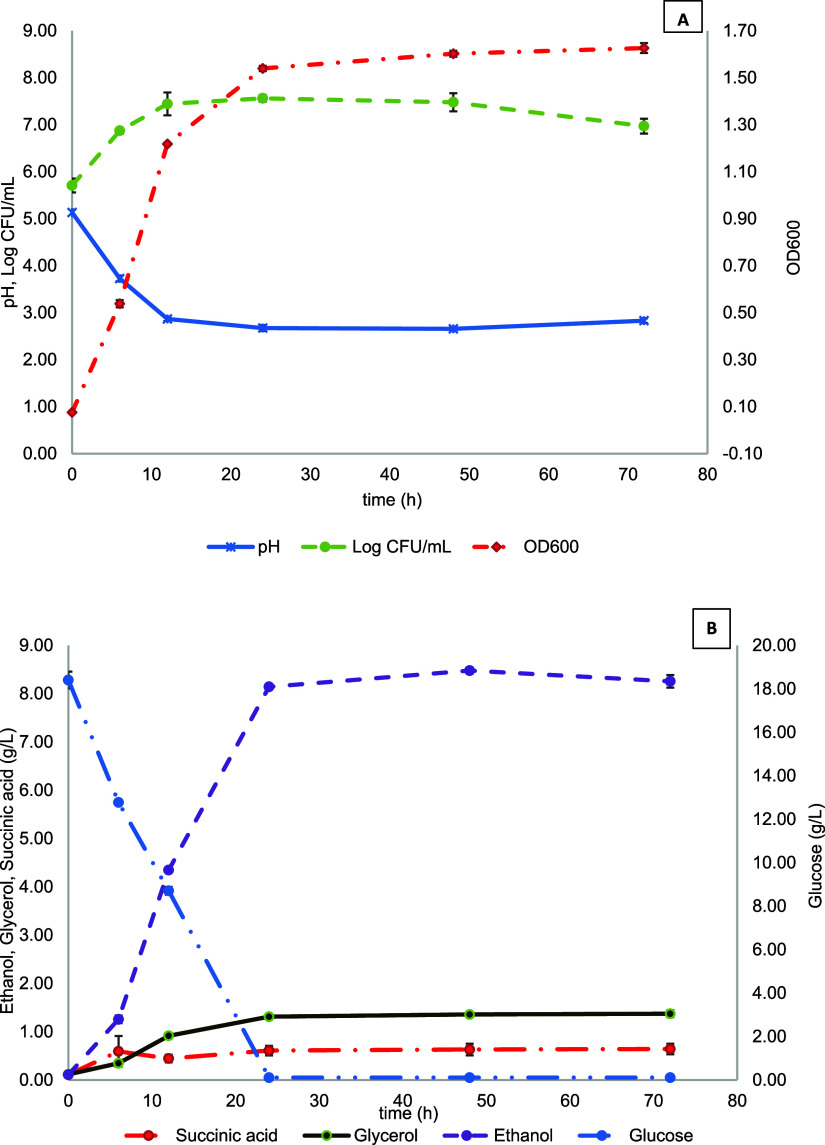
(A) Monitoring of pH, viable yeast (log CFU/mL), and optical density (OD600) during the fermentation of Yeast Nitrogen Base without amino acids (YNBwAA) minimal medium containing 20 g/L dextrose by *S. cerevisiae* OS3. (B) Glucose (g/L) consumption and ethanol (g/L), glycerol (g/L), and succinic acid (g/L) production during the fermentation of Yeast Nitrogen Base without amino acids (YNBwAA) minimal medium containing 20 g/L of dextrose by *S. cerevisiae* OS3.

Contamination from aerobic bacteria during the fermentation process was carefully avoided and checked by analyzing 0.1 mL of each replica batch on PCA and MRS agar, both containing cycloheximide. Moreover, a microscope examination of the fermentation broth did not reveal the presence of any possible bacteria.

### 3.2 Extraction and Identification of Yeast and Bacterial Signaling Molecules

The cell-free supernatant extracts of *S. cerevisiae* OS3 and V5 strains at different growth time points (0, 6, 12, 24, 48, and 72 h for the OS3 strain and 0, 6, and 24 h for the V5 strain) were analyzed by NMR spectroscopy to detect secondary metabolites involved in inter- and intraspecies communication. A comparison with NMR spectra of pure tyrosol, farnesol, 2-phenylethanol, 2-phenoxyethanol, and tryptophol secured the exclusive presence of tyrosol as the fungal QS metabolite recovered in the ethyl acetate extract spectra of the spent medium. In the ^1^H NMR spectra, the aromatic proton signals of tyrosol were easily detected at 7.02 ppm (*doublet*, *J* 8.89 Hz) and 6.69 ppm (*doublet*, *J* 8.89 Hz); the alchilic proton signals (2.71, *triplet*, *J* 7.31 Hz) and 3.67 (*triplet, J* 7.31 Hz) ppm overlapped with other signals and were assigned by homonuclear and heteronuclear 2D experiments and by comparison with spectrum of tyrosol standard (Figures S1–S5). Significant levels of tyrosol were detected in the OS3 strain starting after 6 h of growth (49.53 ± 14.29 nM), which increased over time to 230.58 ± 60.61 nM at 48 h and 309.14 ± 15.45 nM at 72 h (Figure 2). Differently, the tyrosol concentration evaluated in V5 was negligible at 6 h, and of 144.30 ± 15.30 nM at 24 h of growth.

**Figure 2:**
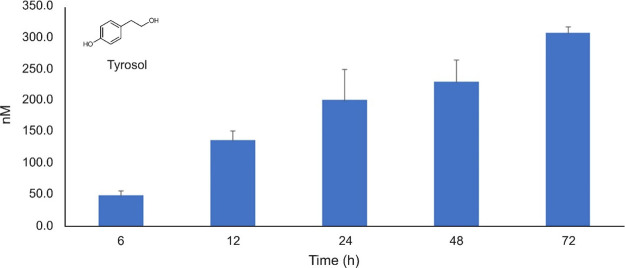
Tyrosol production in the extracellular medium of *S. cerevisiae* OS3 during the fermentation process at different times (starting from 6 to 72 h). Within-sample statistical variation at different time points was evaluated with one-way repeated measures ANOVA with Bonferroni correction. Full results from multiple comparisons are reported in Table S2.

To evaluate possible interference of fungal QSMs with QS system of bacteria, the cell-free medium extracts of *S. cerevisiae* OS3 and V5 strains were analyzed at different time (0, 6, 12, 24, 48, and 72 h for OS3 strain and 0, 6, and 24 h for V5 strain) using the *A. tumefaciens* NTL4 (pZLR4) AHL bioreporter in the TLC-overlay assay. Several extracts were found to activate the AHL bioreporter starting from *t* = 12 h (Figure 3A,B). The first hypothesis was the presence of AHL-mimicking compounds. This behavior has already been described in the literature, where biosensors for AHL detection were activated by diketopiperazines, as shown in the study of Tommonaro et al.^41^ The samples exhibiting positive results in the overlay-assay TLC were analyzed by UPLC-mass spectrometry and, surprisingly, two AHLs were identified, *N*-octanoyl l-homoserine lactone (C8-HSL) and *N*-decanoyl l-homoserine lactone (C10-HSL) (Figures 4 and S6–S8), two typical QSMs produced by bacteria. C8-HSL was the most accumulated molecule; however, the production of both signal molecules increased over time, following the same trend. At *t* = 48 h, there was the maximum production of compounds with concentrations of 0.835 ± 0.287 nM for C8-AHL and 0.174 ± 0.059 nM for C10-AHL (Figure 4).

**Figure 3:**
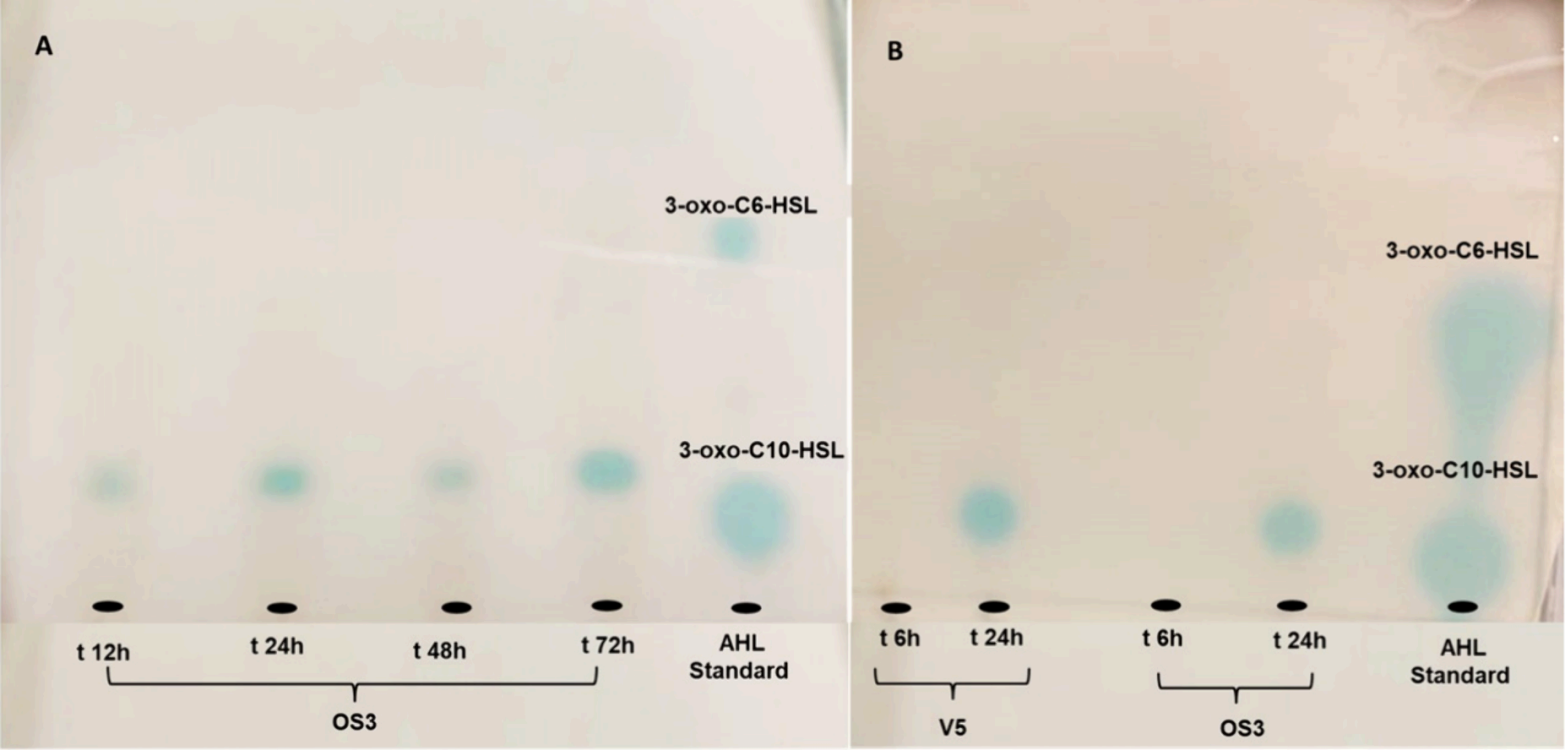
TLC overlay of cell-free medium extracts of (A) *S. cerevisiae* OS3 strain at 12, 24, 48, and 72 h of growth and (B) *S. cerevisiae* V5 strain at 6 and 24 h of growth compared with the OS3 strain. *A. tumefaciens* NTL4 (pZLR4) was used as the bioreporter. AHLs signal molecules were detected by the presence of blue spots. Standards were N-(3-Oxohexanoyl)-l-homoserine lactone (3-oxo-C6-HSL) and N-(3-Oxodecanoyl)-l-homoserine lactone (3-oxo-C10-HSL).

**Figure 4:**
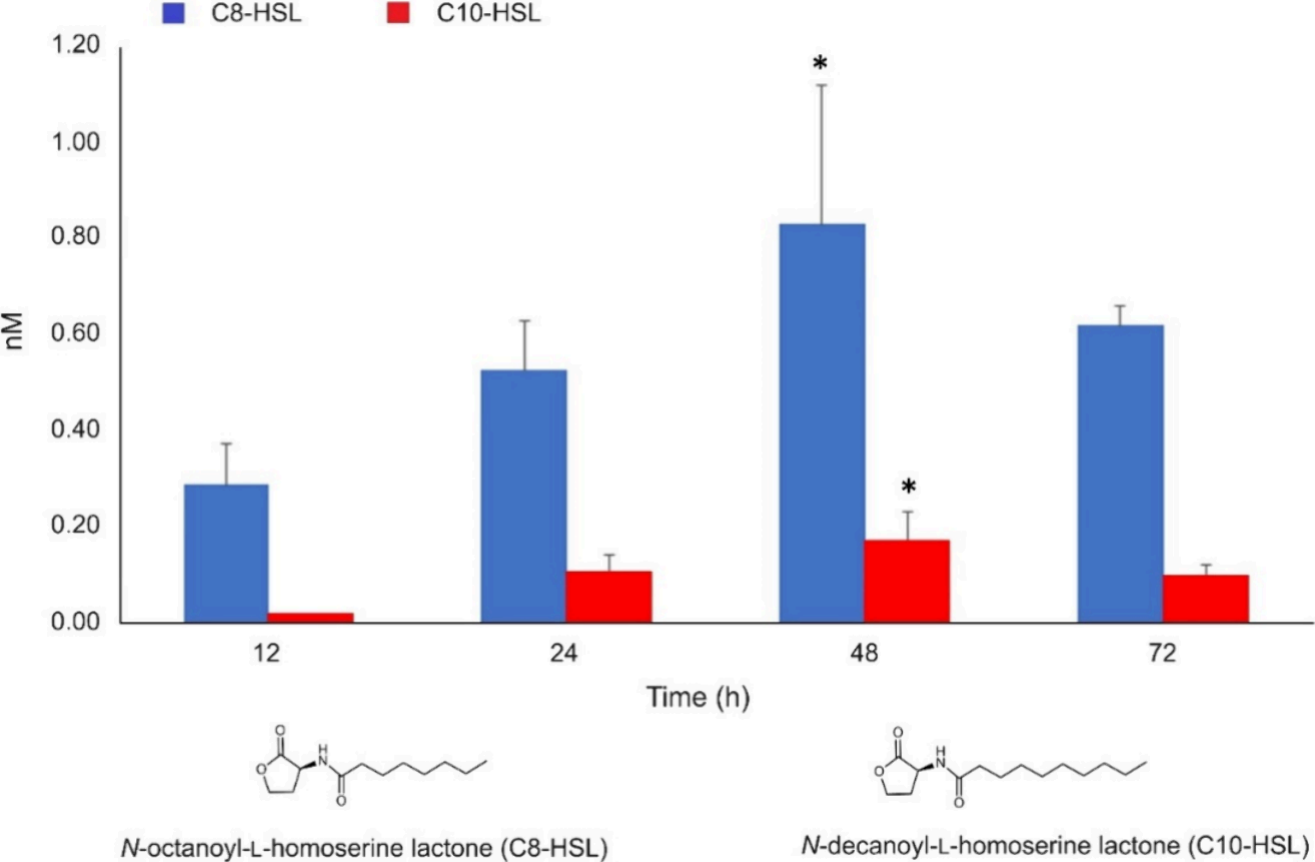
*N*-octanoyl l-homoserine lactone (C8-HSL) and *N*-decanoyl l-homoserine lactone (C10-HSL) production in the extracellular medium of *S. cerevisiae* during the fermentation process. Within-sample statistical variation at different time points was evaluated with one-way repeated measures ANOVA. For both *N*-octanoyl l-homoserine lactone (C8-HSL) and *N*-decanoyl L-homoserine lactone (C10-HSL), only the 12-h versus 48-h comparison showed a statistically significant difference, as reported in the corresponding bar plot (*p-adj < 0.05).

### 3.3 Whole-Genome Analysis, Microscope Observations, and Metataxonomic Analysis

AHL-dependent QS are typically synthesized by a LuxI-type enzyme and detected by LuxR-type transcriptional regulators. We therefore searched for the presence of LuxR (sequence searched: NC_011186.1:1162619-1163371) and acyl-homoserine-lactone synthase in the *S. cerevisiae* OS3 genome with the aim of detecting AHL synthase gene *(luxI*) and a transcriptional regulator gene (*luxR*). For this, we used the Basic Local Alignment Search Tool suite (BLASTN and TBLASTX routines, v. 2.13.0). The alignment of lux genes against the *S. cerevisiae* genome did not match any sequence. Therefore, considering the strictly applied experimental conditions of growth to rule out possible external contamination, the production of AHLs supports the hypothesis of the coexistence of bacteria in *S. cerevisiae* OS3 and V5 strains.

Suspensions of cells cultivated in liquid YPD and in the presporulation media YPA, YPSM, YSM, and BP were analyzed by an optical microscope. The analysis showed MBLBs inside the cells of *S. cerevisiae* OS3 and V5 strains grown on YSM medium, as the bodies occupy different positions in the A and B pictures of Figure 5 (1000× magnification), taken a few seconds apart (red arrows). This is more evident in the Supporting Information, Video S1. By using presporulation YPA medium (stress conditions), the presence of MBLBs became greater and more evident (Figure 5, pictures C and D).

**Figure 5:**
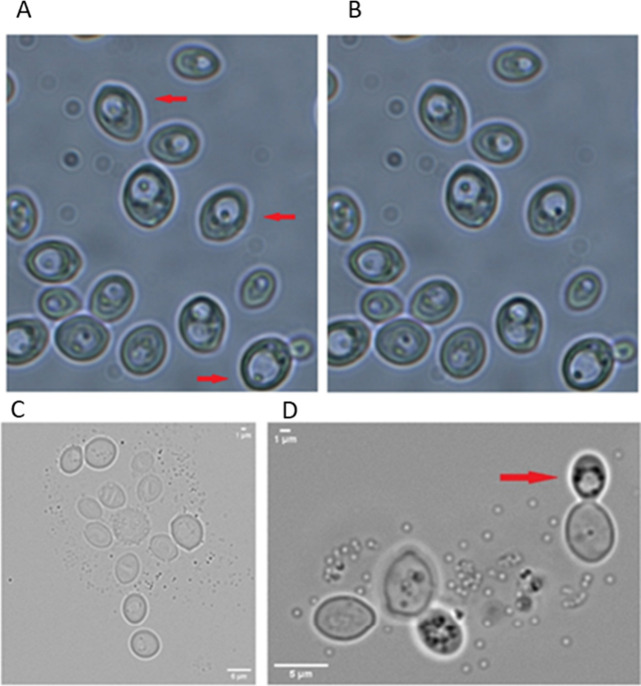
Microscope analysis (1000× magnification) of moving bacteria-like bodies (MBLBs) inside the yeast cell of strain OS3 grown on YSM medium. (A,B) The red arrow in picture A points to the bacteria-like body moving inside the yeast cell. The pictures were taken a few seconds apart to show the active movement of the bacteria-like body. (C) As stress conditions increase (YPA medium 48 h), the presence in OS3 cells of MBLBs becomes greater. The figure shows them that they exit from the yeast cell, becoming empty. (D) The figure shows a cell of OS3 yeast surrounded by bacteria-like bodies (MBLBs) in potassium acetate, 96 h, 30 °C. Under severe stress conditions, caused by the absence of nutrients, the occurrence of dark dots (indicated by the arrow) within the cell can be seen. Dark dots surrounding the vacuole do not move and might consist of stress granules.

After 48 h of growth in YPA medium, as stress conditions increased, MBLBs exited from the OS3 cells (black dots in Figure 5C), leaving them empty. In potassium acetate, after 96 h at 30 °C, under severe stress conditions caused by the absence of nutrients, we observed that the OS3 yeast cells were surrounded by MBLBs, with the occurrence of dark dots (indicated by the red arrow) within the cell. Dark dots surrounding the vacuole do not move and might consist of stress granules. Moreover, the microscopic analysis of YPA-induced *S. cerevisiae* cells of some strains from our collection (including V5 and EC1118) showed, in many cases, the presence of MBLBs inside the vacuoles (data not shown).

Fluorescent microscopy (LIVE/DEAD BacLight) showed the presence of green-stained, small, round, moving BLBs within the yeast’s vacuole (white arrows) of strains OS3 and V5 (Figure S9).

The Sanger sequencing results of amplified 16S rDNA, using DNA isolated from OS3 yeast presenting MBLBs as template, showed confused chromatograms and an overlapping peak, suggesting a mix of 16S rDNA amplicons. This suggested the presence of more than one bacterial species inside the yeast strain, which was further investigated by the Metataxonomic approach (amplicon sequencing). The meta-taxonomic analysis was performed on yeast cultures showing MBLBs, and bacterial communities were assessed by HTS of the amplified V3–V4 regions within the 16S rRNA gene. The main results of two replicas of the strain OS3 are shown in supplementary Figures S10–S13. The core bacterial microbiota retrieved was represented by *Firmicutes*, *Bacteroidota*, and *Proteobacteria* (Figure S10). *Bacteroidaceae, Lachnospiraceae, and Ruminococcaceae* covered about 75% of the population (Figure S11). Particularly, the genus *Bacteroides* with 8 different species was the most occurring (Figures S12 and S13). We also performed several attempts to isolate obligate anaerobic bacteria of the *Bacteroides* group on YCFAGSC, BBA-HVK, and CMM media, but they were resulted unsuccessful.

### 3.4 Enzymatic Analysis

The hydrolytic potentials of the *S. cerevisiae* yeast OS3 and V5 strains were qualitatively investigated. Enzymatic digestion products were qualitatively investigated by TLC (Figure 6), aiming to separate and identify the components of the reaction mixtures, and for each process, the depolymerization degree was evaluated by considering the percentage of the produced glucose equivalent (Table S3, DNS assay).

**Figure 6:**
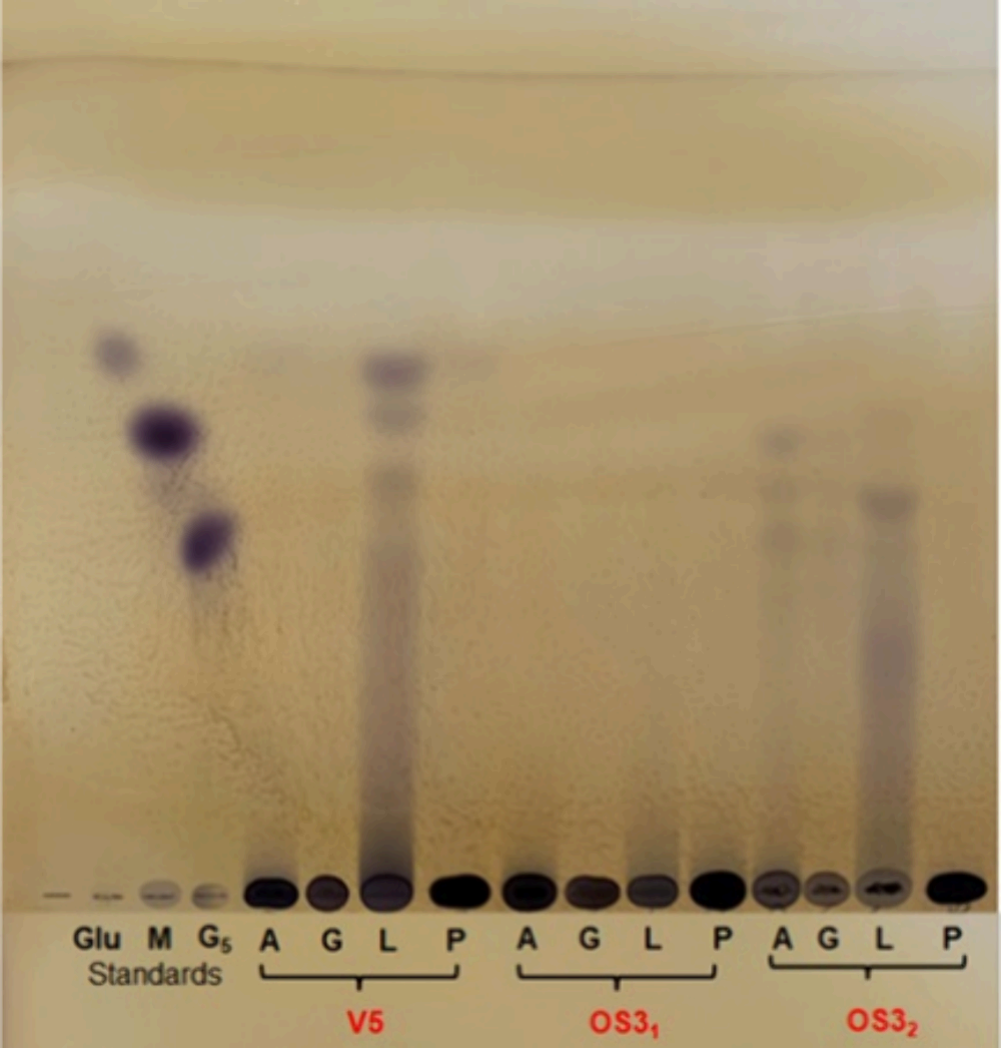
A: Reaction with amylopectin; G: reaction with glycogen; L: reaction with laminarin; P: reaction with pullulan. V5: enzymatic digestion by crude protein enzymatic extracts from *S. cerevisiae* V5 at 24 h of growth in YNBwwAA medium. OS3_1_: enzymatic digestion by crude protein enzymatic extracts from *S. cerevisiae* OS3 at 24 h of growth in YNBwwAA medium. OS3_2_: enzymatic digestion by crude protein enzymatic extracts from *S. cerevisiae* OS3 at 118 h of growth in YSM medium.

These results indicated that in OS3 cells from different growths, among the α-glucans, amylopectin and pullulan were better hydrolyzed (hydrolysis percentage of ∼5:10% by DNS assay) than glycogen; these results suggested the presence in the OS3 cellular system of a pullulanase activity. Pullulanases (EC 3.2.1.41) are well-known starch-debranching enzymes widely used to hydrolyze α-1,6-glucosidic linkages in starch, pullulan, amylopectin, and other oligosaccharides, with application potentials in food, brewing, and pharmaceutical industries.^42^ They were essentially recovered from various bacteria such as *Bacillus* sp., *Thermococcus* sp., *Klebsiella* sp., *Geobacillus* sp.^43^ Differently, the typical isoamylase activity of yeasts was in OS3 and V5 growth conditions, scarcely present^44^ (Figure 6, Table S3).

Among β-1,3-glucans and β-1,3-oligosaccharides, the curdlan, β-glucan from barley, laminarin, and laminaribiose and laminaripentaose were selected for the digestions with the OS3 and V5 crude enzymatic extracts. If curdlan, [a linear polymer consisting of β-(1,3)-linked glucose] and β-glucan from barley, [a linear glucose homopolymer with mixed β(1→3):β(1→4) interglycosidic linkages] were not degraded at all, laminarin, [a glucose polymer made up of β(1→3)-glucan with β(1→6)-branches in the β(1→3):β(1→6) ratio of 3:1] was hydrolyzed by crude enzymatic extracts of OS3 and V5 cells with a percentage of hydrolysis of 9 and 30% (DNS assay), respectively. Oligosaccharides with a degree of polymerization (DP) higher than 5 were produced, and glucose was scarcely detected (Figure 6). It is worth noting that for the OS3 strain under sporulation medium conditions, with increased production of moving bacteria-like bodies, the laminarin digestion was favored. Laminariobiose and laminaripentaose were partially consumed, but glucose was not produced; after 48 h of reactions, oligosaccharides with a DP of 3 or higher were detected (data not shown).

Our results suggest the presence of an endo-1,3-β-glucanase activity and in particular, of a possible laminarinase (EC 3.2.1.39) activity in both OS3 and V5 strains.^45,46^ The characterization of the genetic system governing 1,3-β-glucanase synthesis in yeasts has been reported, even though the exo-1,3-β-glucanase-encoding genes were preferentially detected and investigated with respect to the endo-1,3-β-glucanase-encoding genes.^47,48^

Since the genomes of OS3 and V5 are known, we searched a sequence of pullulanase (X52181.1, 4091 bp long) and laminarase (AB179717.1, 2410 bp long), in the whole set of assemblies from long read sequences from EVOMICS,^34^ and in the described pangenome.^35^ No hits were found in any strain, either with an alignment longer than 200bp, regardless of the identity, or with an alignment length over 25bp and identity over 90%. The absence of genes encoding these enzymes could suggest the bacterial origin of these activities, and these results could represent a further indication of the possible presence of bacteria in the OS3 and V5 yeast cell systems.

## 4 Discussion

Intra- and interspecies interactions between microorganisms are becoming of great interest because of their involvement in several collective microbial behaviors that occur in a microhabitat. In these ecological microniches, communication permits either a cooperative behavior that favors the whole community (strictly communication) or noncooperative behaviors that advantage one or more species over others (i.e., cues or manipulation).^19^ These interactions are based on the production, release, and detection of molecules with different chemical structures, according to the producing microorganism (bacteria, archaea, yeast).^28,41^ QS is the most well-known mechanism of intracellular interaction based on cell-density-dependent chemical signal molecules.

In this paper, we report for *S. cerevisiae* OS3 and V5 strains the chemical identification of tyrosol, QSM of yeasts initially detected as a molecule stimulating the morphological changes and growth of *Candida albicans*,^49^ and for the first time, two QSMs typical of Gram-negative bacteria, C8-HSL and C10-HSL. The latter finding suggested the presence of endosymbiotic bacteria inside *S. cerevisiae* cells, given that yeast cultures were free of external bacteria or any other contamination. Furthermore, since AHL QS-signaling molecules are typically synthesized by LuxI-type enzymes and detected by LuxR-type transcriptional regulators, a search for AHL synthase gene (*luxI*) and a transcriptional regulator gene (*luxR*) in the *S. cerevisiae* strain OS3 genome was performed, but no matches were found. Currently, there is no scientific evidence demonstrating that eukaryotes are capable of directly producing AHLs. However, numerous studies have documented the ability of eukaryotes to detect and respond to these signals, a phenomenon known as “interkingdom signaling.” Moreover, since the synthesis of these molecules is enzymatically regulated and occurs in response to specific physiological conditions of the bacteria, it is completely ruled out that these molecules could arise as byproducts of others.^50–52^ The detection of the QS molecules C8-HSL and C10-HSL in cultures of *S. cerevisiae* OS3 and V5 strains could represent evidence of collective behavior by the bacterial population, leading to gene expression, even though the bacteria are located inside yeast cells. Moreover, yeast cultures were observed with a fluorescent microscope after DNA dyes, with the aim of confirming the cellular location of moving bacteria-like bodies (MBLBs). The presence of enzymatic activities attributable to bacteria hosted in the cellular structure of OS3 and V5 and the absence of genes encoding for them in yeast is a further valuable indication of the existence of intracellular bacteria, which are able to support metabolic activities and fermentation processes. In particular, glucan endo-1,3-β-glucanase enzymes (EC 3.2.1.39) are present in many bacteria of *Bacteroidota* phylum.^53^

These results were in agreement with the metataxonomic analysis on the OS3 strain, indicating a core bacterial microbiota, which was represented by *Firmicutes*, *Bacteroidota*, and *Proteobacteria*. Similar results were recently obtained by analyzing the bacterial communities associated with non-*Saccharomyces* yeasts (*Candida*, *Pichia*, *Meyerozyma*, *Hanseniaspora*, *Rhodotorula*, *Debaryomyces*, *Sporidiobolus*).^1^

However, the difficulties encountered in the cultivation of OS3 endobacteria microbiota could suggest their obligate dependence on the fungal host and may imply that endobacteria complement their metabolism using metabolites from their microbial community. Such a host dependency of fungal endobacteria was recently described in the literature.^54^

Taken together, our results suggest, for the first time, evidence of metabolically active intracellular bacteria in *S. cerevisiae* strains OS3 and V5 cells by a multidisciplinary approach. The bacteria could regulate their gene expression *via* a peculiar signaling pathway by the production and detection of C8-HSL and C10-HSL molecules, indicating control of their behavior, even within yeast cells. The biosynthesis of numerous biomolecules, mainly enzymes, by bacteria is regulated by the production, diffusion, and detection of AIs, predominantly AHLs.^28^ Our data point to the potential existence of a metabolic network that integrates quorum-sensing mechanisms with reported enzymatic activities. Meanwhile, *S. cerevisiae* strain OS3 accumulated extracellular tyrosol in a peculiar concentration range as soon as the cellular density increased.

A similar chemical approach was described in the study conducted by Kai and co-workers^55^ on the zygomycete fungus *Mortierella alpina* A-178. They described the isolation and identification of *N*-heptanoyl-l-homoserine lactone (C7-HSL) and C8-HSL from the culture broth of *M. alpina* A-178. This surprising result led them to perform additional analysis confirming the presence of the endobacterium *Castellaniella defragrans* (sequence identity 100%), which was the true producer of AHLs. However, in recent years, several researchers have discovered important occurrences in which QSMs appear to take on additional roles as interspecies signals that may regulate microbial ecology. A very interesting paper has proved that the budding yeast *S. cerevisiae* responds to the presence of the Gram-negative bacterium *P. aeruginosa*, in particular to its main QS molecule *N*-dodecanoyl-l-homoserine lactone (C12-HSL).^56^ *S. cerevisiae* cells were exposed to a diverse set of QSMs derived from various bacterial species to assess the yeast stress response. Stress levels were quantified by monitoring the expression of Hsp12, a small heat shock protein fused to a green fluorescent protein (GFP). Among the QSMs tested, only the *Pseudomonas*-derived molecule C12-HSL elicited a significant stress response. In contrast, other QSMs, including C4-HSL (from the *P. aeruginosa* RhlI/RhlR system), 3-oxo-C8-HSL (from the *A. tumefaciens* TraI/TraR system), 4,5-dihydroxy-2,3-pentanedione (DPD), and farnesol (a QSM from *C. albicans*), did not induce a stress response above the control levels.

Previously, *S. cerevisiae* has been used as an artificial host for “endobacteria” to establish the endosymbiotic theory of mitochondrial evolution, using engineered *Escherichia coli*, and the yeast strain *S. cerevisiae* W303 was used as an artificial host for *Wolbachia pipientis*.^57–59^ In this study, the authors employed the *S. cerevisiae* W303 strain as an alternative host for *Wolbachia* wAlbB and investigated the resulting host-endosymbiont interaction. Yeast cells harboring the infection exhibited reduced viability compared to uninfected controls, likely due to abnormally elevated mitochondrial oxidative phosphorylation activity observed during the later stages of growth. *Wolbachia* infection resulted in the activation of mitochondria beyond the stationary growth phase. It may be speculated that such activation constitutes an advantage for *W. pipientis* either due to quenching of oxygen in the cytoplasm or because *W. pipientis* needs high ATP that an active mitochondria provides.

It was previously described that aromatic alcohols, tyrosol (TyrOH), tryptophol (TrpOH), and phenylethanol (PheOH) act as QSMs in yeasts, regulating cell density, evoking morphological changes, and playing a crucial role in communication and adaptation to the microhabitat in which they live.^17,60^ For instance, the human fungal pathogen *Candida albicans* exhibits a pronounced growth lag when transferred into fresh minimal medium. This delay is eliminated by supplementing the medium with a conditioned supernatant from a high-density culture. The active factor responsible is tyrosol, a molecule secreted continuously during growth. Under conditions that support germ-tube development, tyrosol promotes the emergence of these filamentous structures. In contrast, germ-tube formation is suppressed by farnesol, which is another QSM, suggesting that this morphogenetic process is tightly regulated by opposing environmental signals. The discovery of tyrosol as an autoregulatory compound underscores its critical role in modulating growth dynamics and morphological transitions in *Candida*.^49^

Regardless of the bacterial core, in the selected growth conditions with Yeast Nitrogen Base medium, *S. cerevisiae* OS3 and V5 accumulated extracellular tyrosol as soon as the cellular density increased. At 72 h of yeast growth, a concentration of 309.14 ± 15.45 nM was recorded for OS3, which was lower than values previously reported.^9,49,60^ In fact, in *S. cerevisiae* ZIM 1927, which was originally isolated from the must of “Malvasia” wine grapes and grown on nitrogen-rich MS300 synthetic must, the tyrosol reached the maximal concentration after 42 h of ∼30 μM.^9^ However, as reported by Gonzalez et al.,^60^ the synthesis of aromatic alcohol depends on glucose, nitrogen, and aromatic amino acid availability in the medium in both *Saccharomyces* and non-*Saccharomyces* yeast strains.

Understanding the mechanisms by which microorganisms engage in interactions, it is fundamental to evaluate their effect on industrial processes (e.g., fermenting wine, leavening bread, or brewing beer) and biotechnological applications. The study on the connection between the production of QS molecules and the biological role of the interactions that occur between yeasts and bacteria (their syntrophy and tolerance) could help to improve fermentation conditions, as well as flavor, by directed endosymbiosis, the development of new cellular systems equipped with novel or synthetic organelles and to produce stable symbiotic chimera*.*

In food fermentation processes, microbial consortia affect the final features of the desired products (aroma, texture, flavor, and shelf life). *S. cerevisiae* is the principal yeast species involved in grape fermentation and plays an important role in the formation of wine aromas, as well as its taste balance.^61^ The distinct wine characteristics are affected by the presence of bacterial and fungal consortia in grape fermentations, which influences the process performance, and by the unique combination of natural factors (climate, soil composition, topography, and local biodiversity) associated with a specific location (*terroir*) that influences the characteristics of agricultural products, especially wine.^62,63^ Wine fermentation is a complex process that involves multiple microbial species. It is not to be ruled out that interesting endosymbiotic yeast–bacteria relationships may arise within these microcommunities, where mutual coexistence could result in an unexpected and distinctive fermentative capacity, an aspect that remains entirely unexplored. Although the presence of endosymbionts in yeasts has already been demonstrated, our findings are innovative because, for the first time, chemical analysis brings out the possible presence of endosymbionts also in *S. cerevisiae*, a yeast with high industrial application potential. Results reported in our paper contribute to the advancement of knowledge about biochemical interactions that take place in microbial consortia during the fermentation process. Our findings pave the way for new technologies based on the development of new fermentation processes capable of fully harnessing the potential of selected *S. cerevisiae* strains and their endosymbiotic bacteria. This will allow for the production of wines with unique and enhanced organoleptic profiles.

The unexpected findings of bacterial QS molecules here identified offer novel insights into the endosymbiotic relationship and highlight its potential biological significance. Further investigations will be crucial to fully elucidate the nature of this coexistence and characterize the underlying metabolic pathways. The engineering of interspecies relationships represents a new frontier in synthetic biology, and the data here reported on the involvement of *S. cerevisiae*, the microorganism at the forefront of the International Synthetic Yeast Genome Project (Sc2.0), imply that our study could play a useful part in the challenges of modern biology.

## Supplementary Material





## Notes

Figures S1–S5: NMR spectra of tyrosol; Figures S6–S8: Representative UPLC-MS/MS (MRM) profiles of *S. cerevisiae* OS3 and V5 strains and of standards; Figure S9: Bright field and fluorescent microscopy (LIVE/DEAD BacLight) of *S. cerevisiae* OS3 strain; Figures S10–S13: Metataxonomic analysis of *S. cerevisiae* OS3 strain; Table S1: Protein concentration in crude enzymatic extracts of *S. cerevisiae* OS3 and V5 strains; Table S2: Statistical analysis with one-way repeated measures ANOVA test and Bonferroni correction; Table S3: Qualitative evaluation of substrates hydrolysis by using glucanase activities in the crude extract of cells of *S. cerevisiae* OS3 and V5 strains (PDF)
Video S1: Moving bacteria-like bodies (MBLBs) inside the cells of *S. cerevisiae* OS3 strain (MP4)

## Abbreviations

QS: quorum sensing
AHLs: acyl-homoserine lactones
C8-HSL: *N*-octanoyl-l-homoserine lactone
C10-HSL: *N*-decanoyl-l-homoserine lactone
UPLC-MS/MS: Ultra Performance Liquid Chromatography Mass Spectrometry/Mass Spectrometry
GRAS: generally regarded as safe
QSMs: quorum sensing molecules
IS: intercellular signaling
AI: AutoInducers
YNBwAA: yeast nitrogen base without amino acids
YDP: yeast peptone dextrose
OD: optical density
PCA: plate count agar
MRS agar: Man, Rogosa, and Sharpe agar
HPLC: high-performance liquid chromatography
WLNA: Wallerstein Laboratory Nutrient Agar
NA: nutrient agar
BP-EYT: Baird Parker Agar Base supplemented with Egg’s Yolk Tellurite Steril Emulsion
PA-CFC: Pseudomonas agar Base with Cetrimide Fusidic Acid and Cefaloridin supplement
YPSM: yeast pre-sporulation medium
YSM: yeast sporulation medium
MBLBs: moving bacteria-like bodies
YCFAGSC medium: yeast casitone fatty acids agar with glucose, starch, and cellobiose
BBA-HVK: Brucella Blood Agar with Hemin and Vitamin K_1_
CMM: chopped meat medium
YPA medium: yeast, peptone potassium acetate
TLC-overlay assay: thin-layer chromatography-overlay assay
TOCSY-, HSQC-, HMBC- NMR spectroscopy: TOtal Correlation SpectroscopY-, heteronuclear single quantum coherence-, heteronuclear multiple bond correlation- nuclear magnetic resonance spectroscopy
^1^H NMR: proton nuclear magnetic resonance
MeOD: Methanol-d4
TMS: TetraMethylSilane
RP-C18 thin-layer chromatography: reversed-phase thin-layer chromatography with a stationary phase consisting of silica particles coated with a C18 alkyl chain
ATGN: *Agrobacterium tumefaciens* Gram negative broth
X-Gal: 5-bromo-4-chloro-3-indolyl-beta-D-galactopyranoside
ACN: ACetoNitrile
MRM: multiple reaction monitoring
PBS: phosphate buffered saline
EtOAc:AcOH:2-Propanol:HCOOH:H_2_O: ethyl acetate, acetic acid: 2-propanol: formic acid: water
DNS assay: 3,5-dinitrosalicylic acid assay
HTS: high-throughput sequencing
CFU: colony forming unit
DP: degree of polymerization

## References

[1] Indu B., Keertana T., Ipsita S., Jagadeeshwari U., Sasikala C., Ramana C. V. (2021). Uncovering the hidden bacterial ghost
communities of yeast and experimental evidences demonstrates yeast
as thriving hub for bacteria. Sci. Rep..

[2] Mares-Rodriguez F. d. J., Aréchiga-Carvajal E. T., Ruiz-Herrera J., Moreno-Jiménez M. R., González-Herrera S. M., León-Ramírez C. G., Martínez-Roldán A. d. J., Rutiaga-Quiñones O. M. (2023). A new bacterial endosymbiotic relationship
in *Kluyveromyces marxianus* isolated from the mezcal
fermentation process. Process Biochem..

[3] Sánchez-Alonzo K., Silva-Mieres F., Arellano-Arriagada L., Parra-Sepúlveda C., Bernasconi H., Smith C. T., Campos V. L., García-Cancino A. (2021). Nutrient Deficiency
Promotes the Entry of *Helicobacter pylori* Cells into *Candida* Yeast Cells. Biology.

[4] Tavakolian A., Heydari S., Siavoshi F., Brojeni G. N., Sarrafnejad A., Eftekhar F., Khormali M. (2019). Localization
of *Staphylococcus* inside the vacuole of *Candida
albicans* by immunodetection
and FISH. Inf. Gen. Evol..

[5] Kang S. W., Jeon B. Y., Hwang T. S., Park D. H. (2009). Symbiotic relationship
between *Microbacterium* sp. SK0812 and *Candida
tropicalis* SK090404. J. Microbiol..

[6] U.S. Food & Drugs. https://www.fda.gov/food/gras-notice-inventory/recently-published-gras-notices-and-fda-letters (accessed 2024-January-20).

[7] Liti G. (2015). The Natural
History of Model Organisms: The fascinating and secret wild life of
the budding yeast *S. cerevisiae*. eLife.

[8] Pretorius I. S. (2017). Synthetic
genome engineering forging new frontiers for wine yeast. Crit. Rev. Biotechnol..

[9] Avbelj M., Zupan J., Raspor P. (2016). Quorum-sensing
in yeast and its potential
in wine making. Appl. Microbiol. Biotechnol..

[10] Miller M., Bassler B. (2001). Quorum sensing in bacteria. Annu.
Rev. Microbiol..

[11] Winzer K., Hardie K. R., Williams P. (2002). Bacterial
cell-to-cell communication:
sorry, can’t talk now—gone to lunch!. Curr. Opin. Microbiol..

[12] Abbamondi G. R., Tommonaro G. (2022). Research Progress and Hopeful Strategies
of Application
of Quorum Sensing in Food, Agriculture and Nanomedicine. Microorganisms.

[13] Barriuso J., Hogan D. A., Keshavarz T., Martínez M. J. (2018). Role of
quorum sensing and chemical communication in fungal biotechnology
and pathogenesis. FEMS Microbiol. Rev..

[14] Zhang L., Li S., Liu X., Wang Z., Jiang M., Wang R., Xie L., Liu Q., Xie X., Shang D., Li M., Wei Z., Wang Y., Fan C., Luo Z. Q., Shen X. (2020). Sensing of
autoinducer-2 by functionally distinct receptors in prokaryotes. Nat. Commun..

[15] Egbe N. E., Dornelles T. O., Paget C. M., Castelli L. M., Ashe M. P. (2017). Farnesol
inhibits translation to limit growth and filamentation in *C. albicans* and *S. cerevisiae*. *Microb*.. Cell.

[16] Polke M., Sprenger M., Scherlach K., Albán-Proaño M. C., Martin R., Hertweck C., Hube B., Jacobsen I. D. (2017). A functional
link between hyphal maintenance and quorum sensing in *Candida
albicans*. Mol. Microbiol..

[17] Chen H., Fink G. R. (2006). Feedback control
of morphogenesis in fungi by aromatic
alcohols. Genes Dev..

[18] Gori K., Knudsen P. B., Nielsen K. F., Arneborg N., Jespersen L. (2011). Alcohol-based
quorum sensing plays a role in adhesion and sliding motility of the
yeast *Debaryomyces hansenii*. FEMS Yeast Res..

[19] Winters M., Arneborg N., Appels R., Howell K. (2019). Can community-based
signalling behaviour in *Saccharomyces cerevisiae* be
called quorum sensing? A critical review of the literature. FEMS Yeast Res..

[20] Chauhan N. M., Mohan Karuppayil S. (2021). Dual identities for various alcohols in two different
yeasts. Mycology.

[21] Zupan J., Avbelj M., Butinar B., Kosel J., Sergan M., Raspor P. (2013). Monitoring of quorum-sensing
molecules during minifermentation
studies in wine yeast. J. Agric. Food Chem..

[22] González-Marco A., Jiménez-Moreno N., Ancín-Azpilicueta C. (2010). Influence
of nutrients addition to nonlimited-in-nitrogen must on wine volatile
composition. J. Food Sci..

[23] Wang H., Dong Q., Guan A., Meng C., Shi X., Guo Y. (2011). Synergistic inhibition
effect of 2-phenylethanol and ethanol on bioproduction
of natural 2-phenylethanol by *Saccharomyces cerevisiae* and process enhancement. J. Biosci. Bioeng..

[24] Yue J. X., Li J., Aigrain L., Hallin J., Persson K., Oliver K., Bergström A., Coupland P., Warringer J., Lagomarsino M. C., Fischer G., Durbin R., Liti G. (2017). Contrasting
evolutionary genome dynamics between domesticated and wild yeasts. Nat. Genet..

[25] Aponte M., Blaiotta G. (2016). Selection of an autochthonous *Saccharomyces
cerevisiae* strain for the vinification of “Moscato
di Saracena”, a southern Italy (Calabria Region) passito wine. Food Microbiol..

[26] Lopez-Siles M., Khan T. M., Duncan S. H., Harmsen H. J. M., Garcia-Gil L. J., Flint H. J. (2012). Cultured Representatives
of Two Major Phylogroups of
Human Colonic *Faecalibacterium prausnitzii* Can Utilize
Pectin, Uronic Acids, and Host-Derived Substrates for Growth. Appl. Environ. Microbiol..

[27] DSMZ, German Collection of Microorganisms and Cell Cultures GmbH https://www.dsmz.de/microorganisms/medium/pdf/DSMZ_Medium78.pdf. (accessed on 2024-January-22).

[28] Tramice A., Cutignano A., Iodice A., Poli A., Finore I., Tommonaro G. (2021). Involvement of a Quorum Sensing Signal Molecule in
the Extracellular Amylase Activity of the Thermophilic *Anoxybacillus
amylolyticus*. Microorganisms.

[29] Tempé J., Petit A., Holsters M., Montagu M. v., Schell J. (1977). Thermosensitive
step associated with transfer of the Ti plasmid during conjugation:
possible relation to transformation in crown gall. Proc. Natl. Acad. Sci. U. S. A..

[30] Steindler L., Venturi V. (2007). Detection of quorum-sensing N -acyl homoserine lactone
signal molecules by bacterial biosensors. FEMS
Microbiol. Lett..

[31] Abbamondi G. R., Suner S., Cutignano A., Grauso L., Nicolaus B., Toksoy Oner E., Tommonaro G. (2016). Identification of N-Hexadecanoyl-L-homoserine
lactone (C16-AHL) as signal molecule in halophilic bacterium *Halomonas smyrnensis* AAD6. Ann. Microbiol..

[32] Bradford M. M. (1976). A rapid
and sensitive method for the quantitation of microgram quantities
of protein utilizing the principle of protein-dye binding. Anal. Biochem..

[33] Bernfeld, P. Amylases α and β. In Methods in Enzymology; Kaplan, N. ; Colowick, S. , Eds.; Academic Press: New York, NY, USA, 1955; Vol. 1, pp. 149–158.

[34] Miao Z., Ren Y., Tarabini A., Yang L., Li H., Ye C., Liti G., Fischer G., Li J., Yue J. (2025). ScRAPdb: an
integrated pan-omics database for the Saccharomyces cerevisiae reference
assembly panel. Nucleic Acids Res..

[35] Peter J., De Chiara M., Friedrich A., Yue J. X., Pflieger D., Bergström A., Sigwalt A., Barre B., Freel K., Llored A., Cruaud C., Labadie K., Aury J. M., Istace B., Lebrigand K., Barbry P., Engelen S., Lemainque A., Wincker P., Liti G., Schacherer J. (2018). Genome evolution
across 1,011 *Saccharomyces cerevisiae* isolates. Nature.

[36] Aponte M., Esposito F., Sequino G., Blaiotta G., De Filippis F. (2022). Stuck or sluggish
fermentations in home-made beers: Beyond the surface. Int. J. Food Microbiol..

[37] Magoč T., Salzberg S. L. (2011). FLASH: fast length
adjustment of short reads to improve
genome assemblies. Bioinformatics.

[38] Callahan B. J., McMurdie P. J., Rosen M. J., Han A. W., Johnson A. J., Holmes S. P. (2016). DADA2: High-resolution
sample inference from Illumina
amplicon data. Nat. Methods.

[39] O’Donnell S., Yue J. X., Saada O. A., Agier N., Caradec C., Cokelaer T., De Chiara M., Delmas S., Dutreux F., Fournier T., Friedrich A., Kornobis E., Li J., Miao Z., Tattini L., Schacherer J., Liti G., Fischer G. (2023). Telomere-to-telomere
assemblies of
142 strains characterize the genome structural landscape in *Saccharomyces cerevisiae*. Nat. Genet..

[40] R Project for Statistical Computing. https://www.r-project.org/ (accessed on 2024-January-18).

[41] Tommonaro G., Abbamondi G. R., Iodice C., Tait K., De Rosa S. (2012). Diketopiperazines
Produced by the Halophilic Archaeon, *Haloterrigena hispanica* Activate AHL. Bioreporters. Microb. Ecol..

[42] Wang X., Nie Y., Xu Y. (2019). Industrially
produced pullulanases with thermostability:
Discovery, engineering, and heterologous expression. Biores. Technol..

[43] Hii S. L., Tan J. S., Ling T. C., Ariff A. B. (2012). Pullulanase: role
in starch hydrolysis and potential industrial applications. Enzyme Res..

[44] Van
Der Maarel M. J. E. C., Van Der Veen B., Uitdehaag J. C. M., Leemhuis H., Dijkhuizen L. (2002). Properties and applications of starch-converting
enzymes of the α-amylase family. J. Biotechnol..

[45] Gavande, P. V. ; Goyal, A. ; Chapter 6—Endo-β-1,3-glucanase. Editor(s): Arun Goyal, Kedar Sharma, In Foundations and Frontiers in Enzymology, Glycoside Hydrolases, Academic Press, 2023; pp 121–133.

[46] Liberato M. V., Teixeira Prates E., Gonçalves T. A., Bernardes A., Vilela N., Fattori J., Ematsu G. C., Chinaglia M., Machi Gomes E. R., Migliorini Figueira A.
C., Damasio A., Polikarpov I., Skaf M. S., Squina F. M. (2021). Insights into the
dual cleavage activity of the GH16 laminarinase enzyme class on β-1,3
and β-1,4 glycosidic bonds. J. Biol. Chem..

[47] Rodríguez-Peña J. M., Cid V. J., Arroyo J., Nombela C. (2000). A novel family of cell
wall-related proteins regulated differently during the yeast life
cycle. Mol. Cell. Biol..

[48] Baladrón V., Ufano S., Dueñas E., Martín-Cuadrado A. B., del Rey F., Vázquez
de Aldana C. R. (2002). Eng1p, an Endo-1,3-β-Glucanase
Localized at the Daughter Side of the Septum, Is Involved in Cell
Separation in *Saccharomyces cerevisiae*. Eukaryotic Cell.

[49] Chen H., Fujita M., Feng Q., Clardy J., Fink G. R. (2004). tyrosol
is a quorum-sensing molecule in *Candida albicans*. Proc. Natl. Acad. Sci. U.S.A..

[50] Coquant G., Grill J. P., Seksik P. (2020). Impact of
N-Acyl-Homoserine Lactones,
Quorum Sensing Molecules, on Gut Immunity. Front.
Immunol..

[51] Mathesius U., Mulders S., Gao M., Teplitski M., Caetano-Anollés G., Rolfe B. G., Bauer W. D. (2003). Extensive
and Specific Responses of a Eukaryote to Bacterial Quorum-Sensing
Signals. Proc. Natl. Acad. Sci. U.S.A..

[52] Liu L., Zeng X., Zheng J., Zou Y., Qiu S., Dai Y. (2022). AHL-Mediated
Quorum Sensing to Regulate Bacterial Substance and Energy
Metabolism: A Review. Microbiol. Res..

[53] GenomeNet. https://www.genome.jp/dbget-bin/www_bget?ec:3.2.1.39 (accessed on 2024-March-20).

[54] Bonfante P., Desirò A. (2017). Who lives in a fungus? The diversity, origins and functions
of fungal endobacteria living in Mucoromycota. ISME Journal.

[55] Kai K., Furuyabu K., Tani A., Hayashi H. (2012). Production of the quorum-sensing
molecules N-acylhomoserine lactones by endobacteria associated with *Mortierella alpina* A-178. Chembiochem..

[56] Delago A., Gregor R., Dubinsky L., Dandela R., Hendler A., Krief P., Rayo J., Aharoni A., Meijler M. M. (2021). A Bacterial
Quorum Sensing Molecule Elicits a General Stress Response in *Saccharomyces cerevisiae*. Front. Microbiol..

[57] Meaney R. S., Hamadache S., Soltysiak M. P. M., Karas B. J. (2020). Designer endosymbionts:
Converting free-living bacteria into organelles. Cur. Opin. Syst. Biol..

[58] Mehta A. P., Supekova L., Chen J. H., Pestonjamasp K., Webster P., Ko Y., Henderson S. C., McDermott G., Supek F., Schultz P. G. (2018). Engineering yeast
endosymbionts as a step toward the evolution of mitochondria. Proc. Natl. Acad. Sci. U. S. A..

[59] Uribe-Alvarez C., Chiquete-Félix N., Morales-García L., Bohórquez-Hernández A., Delgado-Buenrostro N. L., Vaca L., Peña A., Uribe-Carvajal S. (2019). *Wolbachia
pipientis* grows in *Saccharomyces cerevisiae* evoking early death of the host and deregulation of mitochondrial
metabolism. Microbiol. Open.

[60] Gonzalez B., Vazquez J., Morcillo-Parra M. A., Mas A., Torija M. J., Beltran G. (2018). The production of aromatic alcohols in non-*Saccharomyces* wine yeast is modulated by nutrient availability. Food Microbiol..

[61] Bokulich N. A., Collins T. S., Masarweh C., Allen G., Heymann H., Ebeler S. E., Mills D. A., Lindow S. E. (2016). Associations
among
wine grape microbiome, metabolome, and fermentation behavior suggest
microbial contribution to regional wine characteristics. mBio.

[62] Knight S., Klaere S., Fedrizzi B., Goddard M. R. (2015). Regional microbial
signatures positively correlate with differential wine phenotypes:
evidence for a microbial aspect to terroir. Sci. Rep..

[63] Swiegers J. H., Bartowsky E. J., Henschke P. A., Pretorius I. S. (2005). Yeast and
bacterial modulation of wine aroma and flavour. Aust. J. Grape Wine Res..

