# Supplementary material for: First Evidence of Metabolically Active Intracellular Bacteria in Saccharomyces cerevisiae
Source: J Agric Food Chem. 2025 Oct 7;73(41):26220–31. doi: 10.1021/acs.jafc.5c04823 (PMC12532280; doi:10.1021/acs.jafc.5c04823)
Supplement: Supplementary file 1 [file jf5c04823_si_001.pdf]

First evidence of metabolically active intracellular bacteria in *Saccharomyces cerevisiae*

Annabella Tramice[1], Gianni Liti[2], Annalaura Iodice[1], Gennaro Roberto Abbamondi[1], Federica Carlea[2], Ernesto Petruzziello[3], Adele Cutignano[1], Debora Paris[1], Carmine Iodice[1], Matteo De Chiara[2], Maria Aponte[3], Francesca De Filippis[3,4], Chiara Vischioni[2], Andrea Motta[1], Giuseppe Blaiotta[3,4‡], Giuseppina Tommonaro[1‡]*

[1]Institute of Biomolecular Chemistry, Consiglio Nazionale delle Ricerche, 80078 Pozzuoli (NA), Italy (annabella.tramice@cnr.it; annalauraiodice@cnr.it; gennaroroberto.abbamondi@cnr.it; adele.cutignano@cnr.it; debora.paris@cnr.it; carmine.iodice@cnr.it; andrea.motta@cnr.it)

[2]Université Côte d'Azur, CNRS, INSERM, IRCAN, 06107 Nice Cedex 2, France (liti.gianni@gmail.com; fedecarlea@gmail.com; matnamo@gmail.com; chiara.vischioni@unice.fr)

[3]Department of Agricultural Sciences, Federico II University of Naples, 80055 Portici (NA), Italy (ernesto.petruzziello@unina.it; maria.aponte@unina.it; francesca.defilippis@unina.it; giuseppe.blaiotta@unina.it)

[4]Task Force on Microbiome Studies, Federico II University of Naples, 80126 Naples, Italy.

*Corresponding author: giuseppina.tommonaro@cnr.it

[‡]These authors jointly directed the work

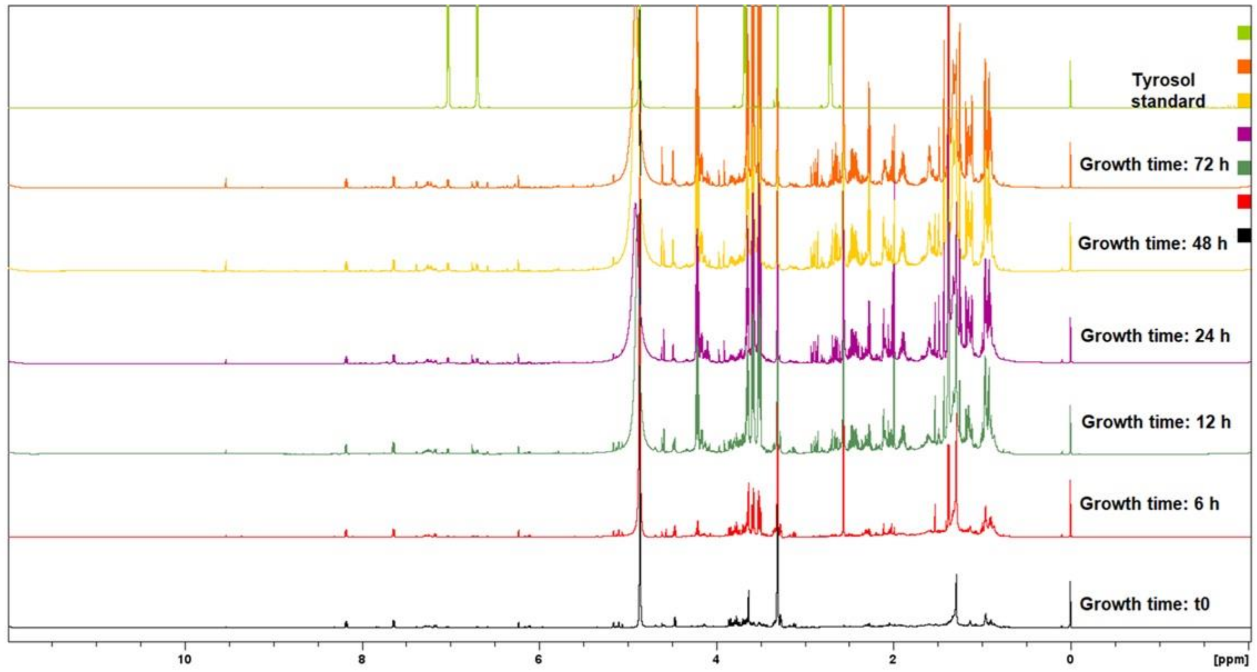

Figure S1

Figure S1. ^{1}H NMR spectra of ethyl acetate extracts of cell-free spent medium from *S. cerevisiae* OS3 strain cultures at different growth stages (t = 0, 6, 12, 24, 48 and 72 h). 5-25 mg of ethyl acetate extracts or 5mg of tyrosol standard were dissolved in 0.700 mL of MeOD containing 0.03% v/v of TMS (Tetramethylsilane, 136.08 µg for each experiment used as internal standard) and the solutions were placed in a 5-mm NMR tube.

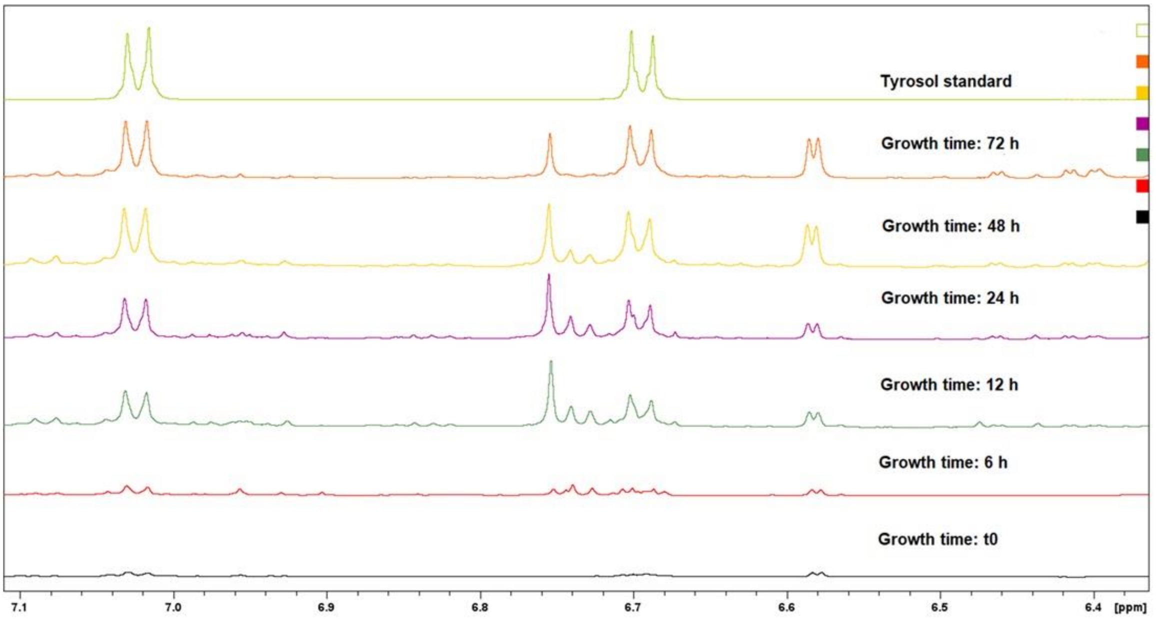

Figure S2

Figure S2. Partial plot of ^{1}H NMR spectra (from 6.3 to 7,15 ppm) of ethyl acetate extracts of cell-free spent medium from *S. cerevisiae* cultures OS3 at different growth stages (t = 0, 6, 12, 24, 48 and 72 h). 5-25 mg of ethyl acetate extracts or 5mg of tyrosol standard were dissolved in 0.700 mL of MeOD containing 0.03% v/v of TMS (Tetramethylsilane, 136.08 µg for each experiment used as internal standard). The monitoring of the increase of the aromatic signals of tyrosol at 7.02 ppm and 6,71 at different time of growth is reported.

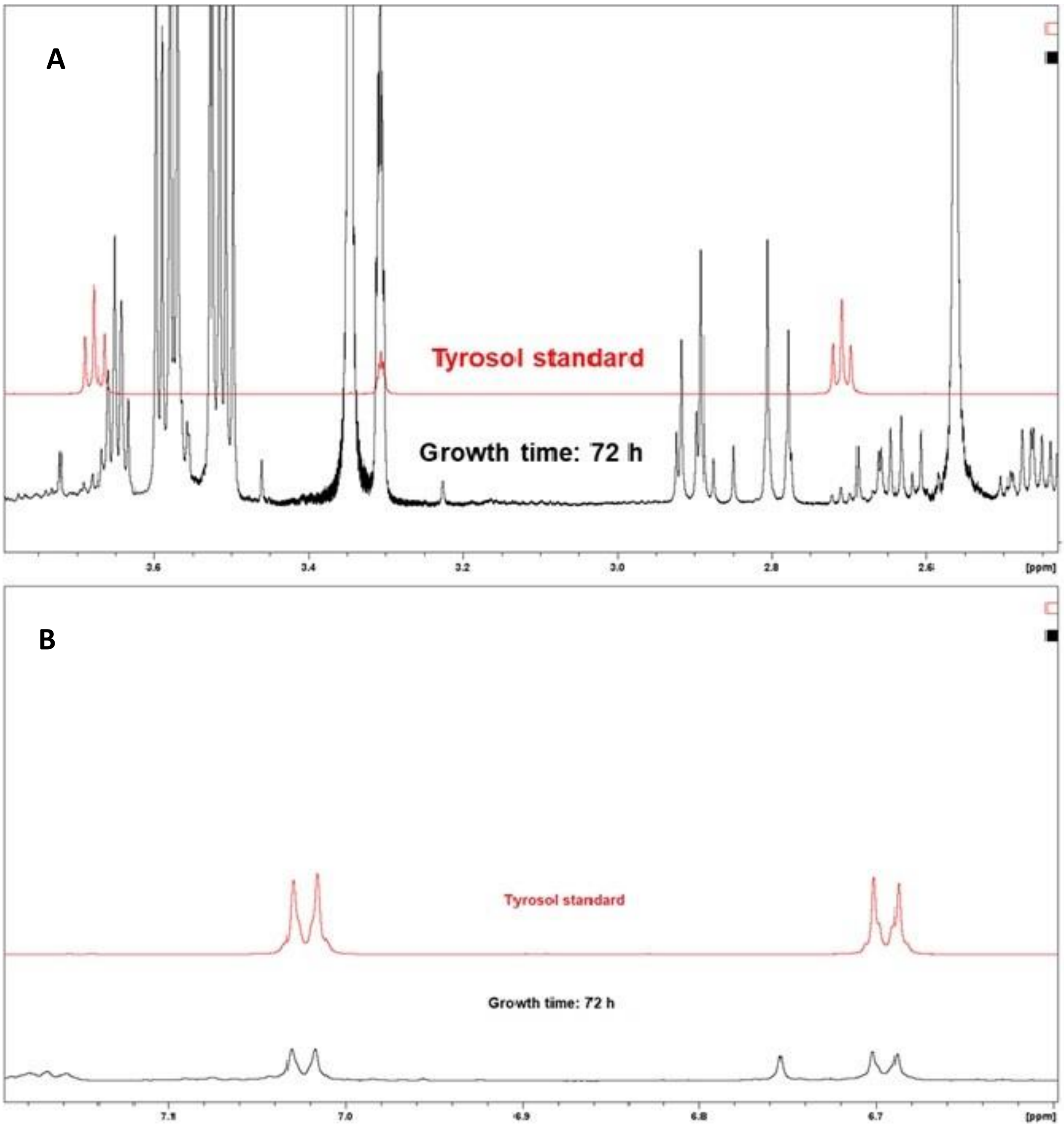

Figure S3. Partial plot of ^{1}H NMR spectra of ethyl acetate extract from *S. cerevisiae* cultures OS3 at 72h of growth (black line) overlapped with tyrosol standard spectrum (red line) in alchilic (S3-A) and aromatic regions (S3-B).

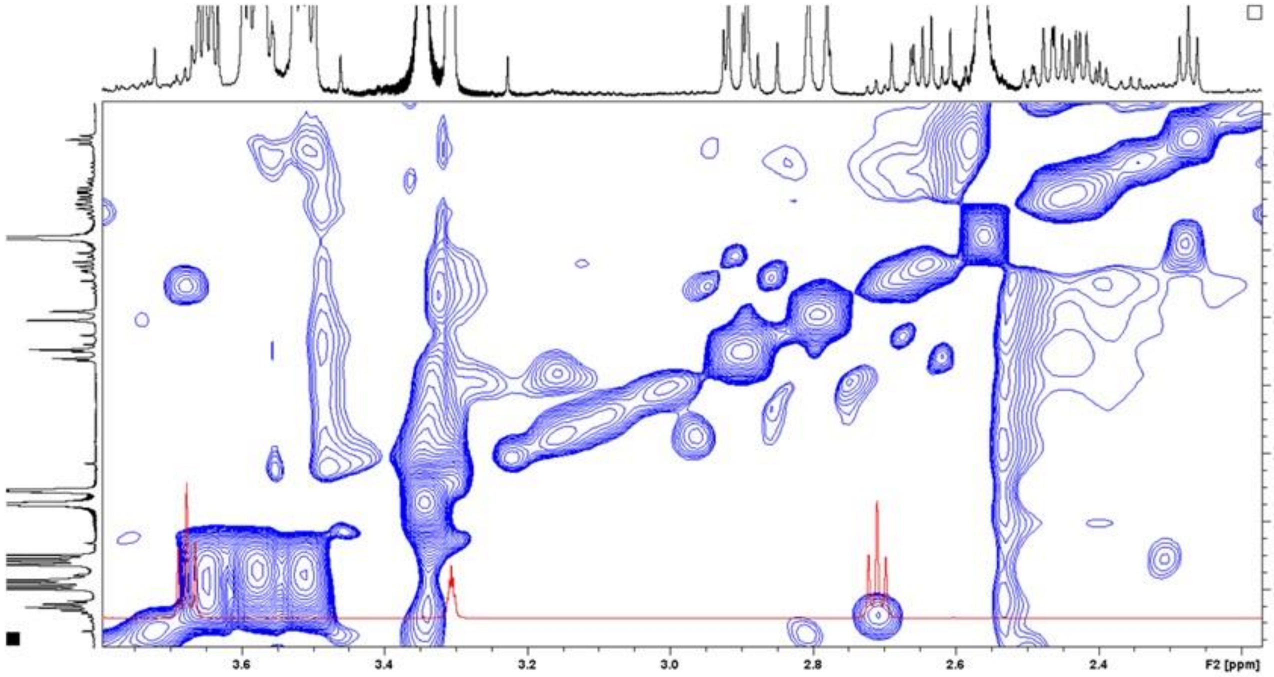

Figure S4

Figure S4. Partial plot of TOCSY-NMR spectrum of ethyl acetate extract from *S. cerevisiae* cultures OS3 at 72h of growth (blue spots) overlapped with ^{1}H NMR spectra with tyrosol standard spectrum (line red) in alchilic regions. Correlation spots off-diagonal are selected (line yellow) secure the alchilic portion of tyrosol presence in OS3 extract

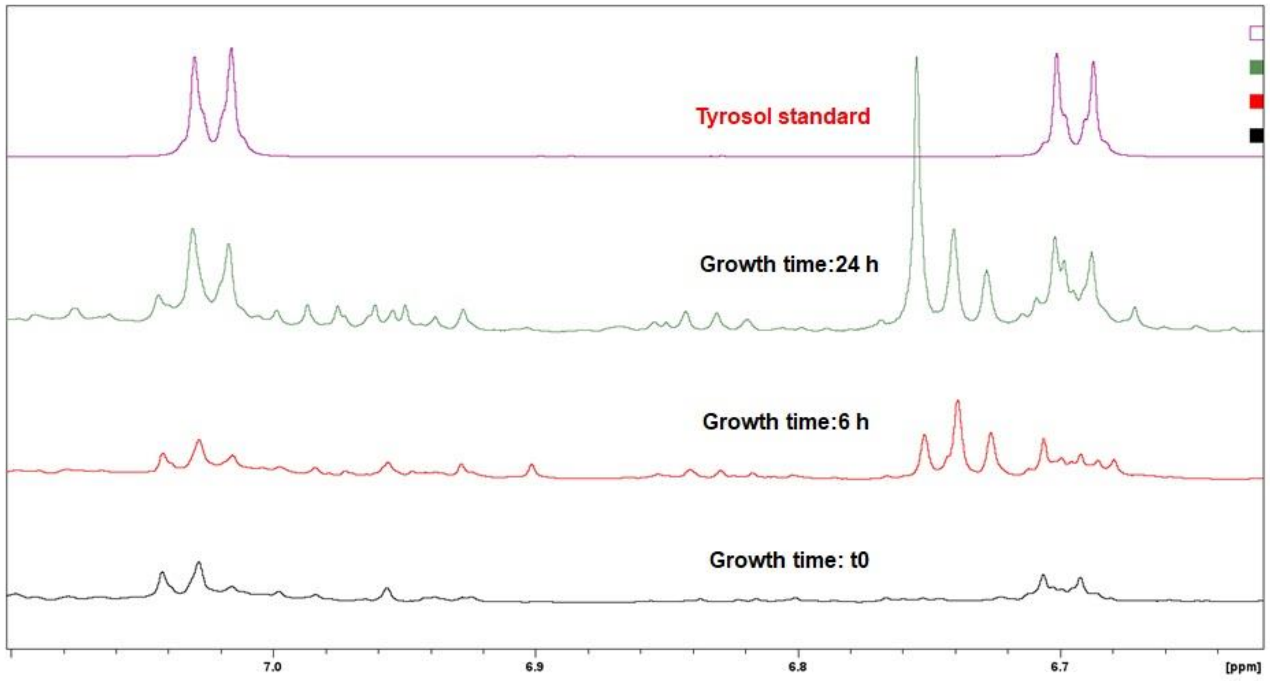

Figure S5. Partial plot of ^1H NMR spectra (from 6.3 to 7,15 ppm) of ethyl acetate extracts of cell-free spent medium from *S. cerevisiae* cultures V5 at different growth stages (t = 0, 6 and 24 h). 5-25 mg of ethyl acetate extracts or 5mg of tyrosol standard were dissolved in 0.700 mL of MeOD containing 0.03% v/v of TMS (Tetramethylsilane, 136.08 μg for each experiment used as internal standard). The monitoring of the increase of the aromatic signals of tyrosol at 7.02 ppm and 6,71 at different time of growth is reported.

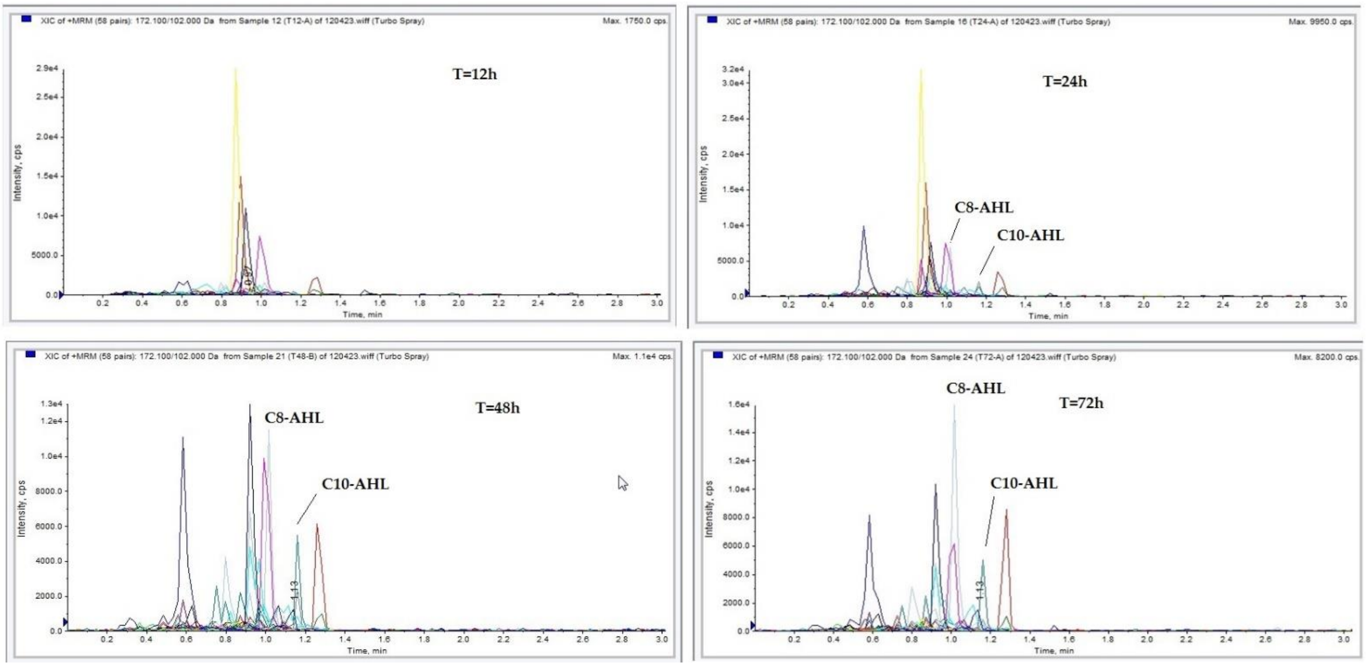

Figure S6. Representative UPLC-MS/MS (MRM) profiles of *S. cerevisiae* OS3 strain at T=12, 24, 48 and 72h growth time.

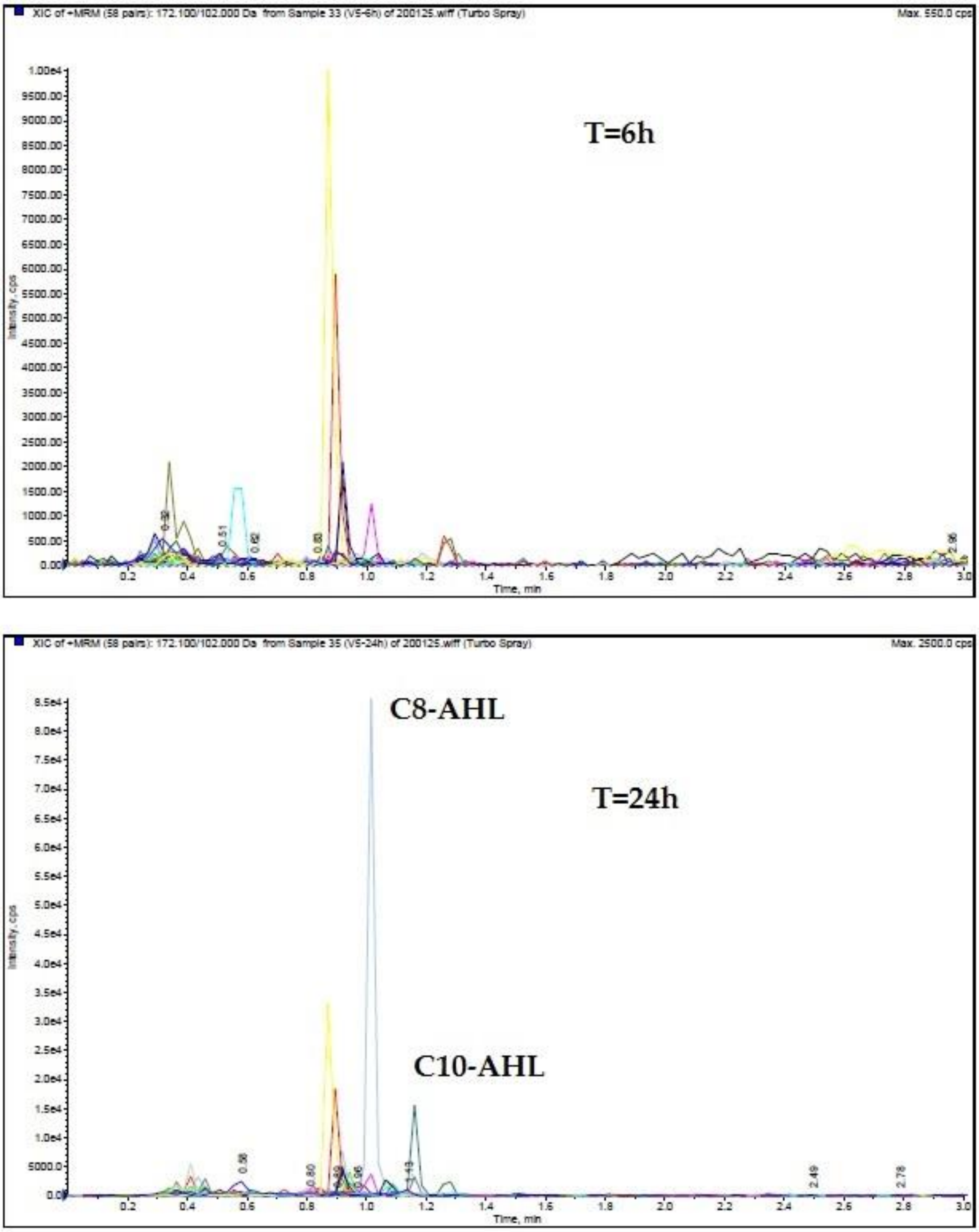

Figure S7. Representative UPLC-MS/MS(MRM) profiles of *S. cerevisiae* V5 strain at T=6 and 24h growth time.

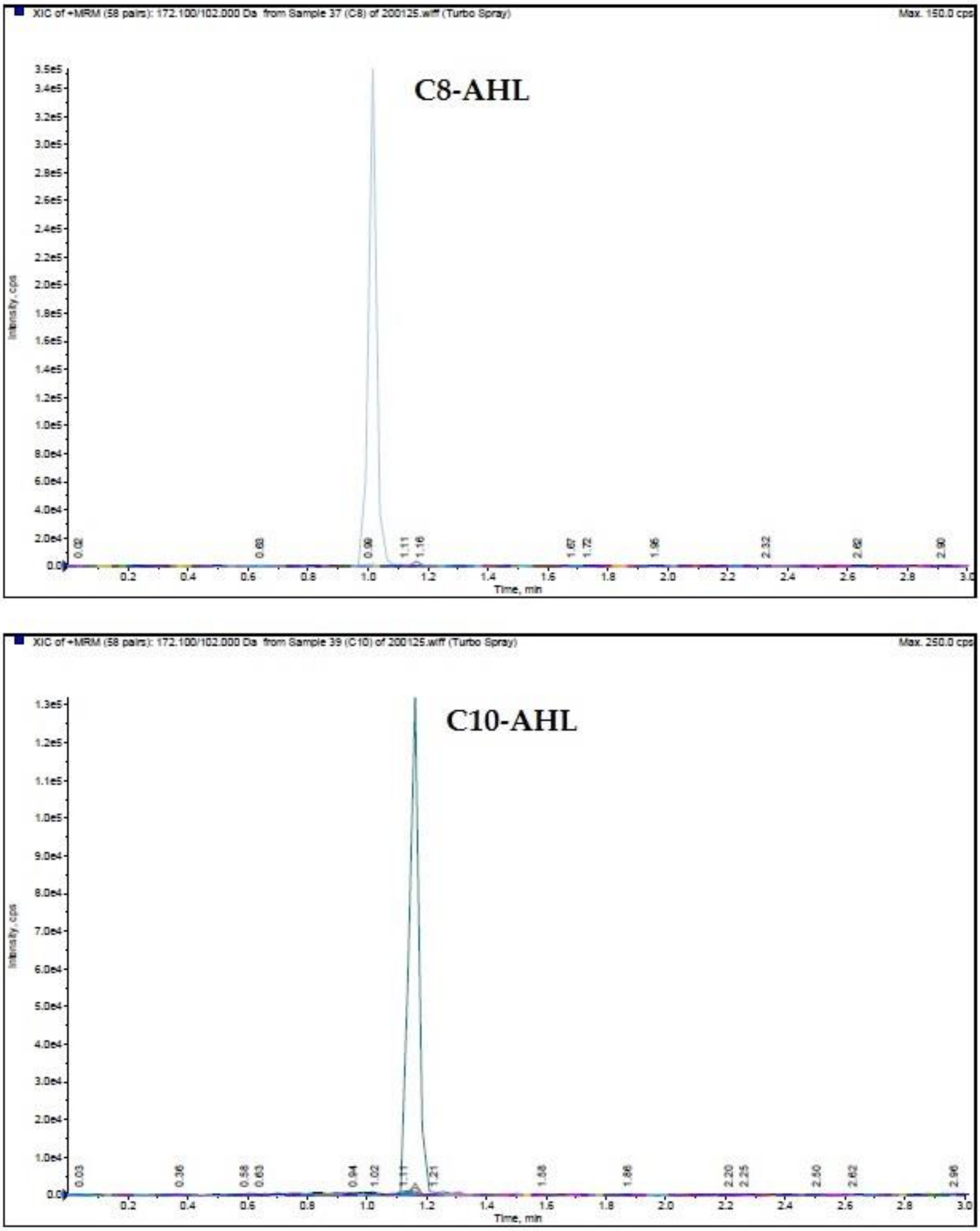

Figure S8. UPLC-MS/MS (MRM) trace of C8-AHL (228>102 m/z) and C10-AHL synthetic standards (256>102 m/z).

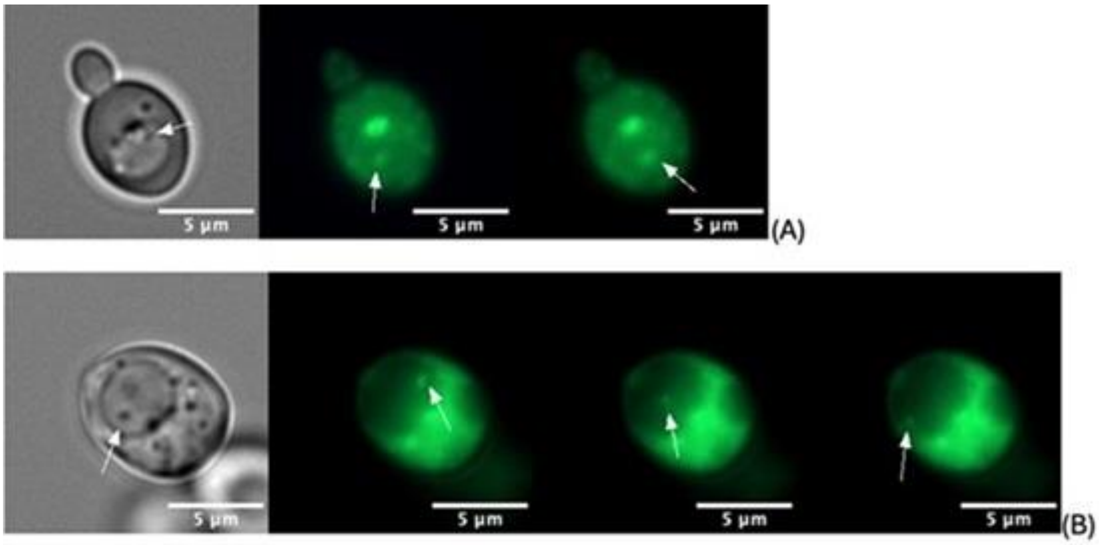

Figure S9. Bright field and fluorescent microscopy (LIVE/DEAD™ BacLight™) of *S. cerevisiae* strains OS3 (panel A) and V5 (panel B). Pictures were taken a few seconds apart to visualize the frequent movements of the MBLBs.

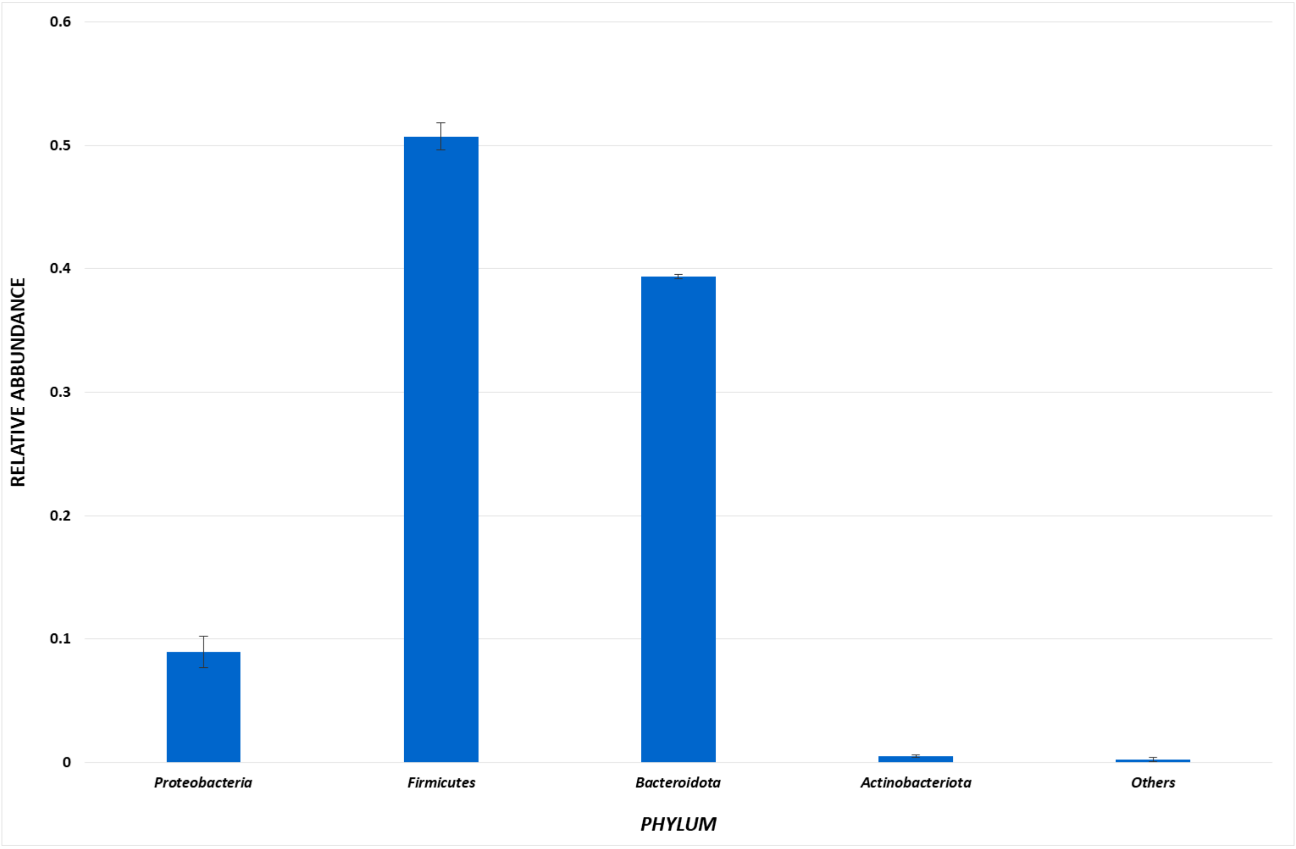

Figure S10. Relative abundance of phylum of the core bacterial microbiota of *S. cerevisiae* strain OS3.

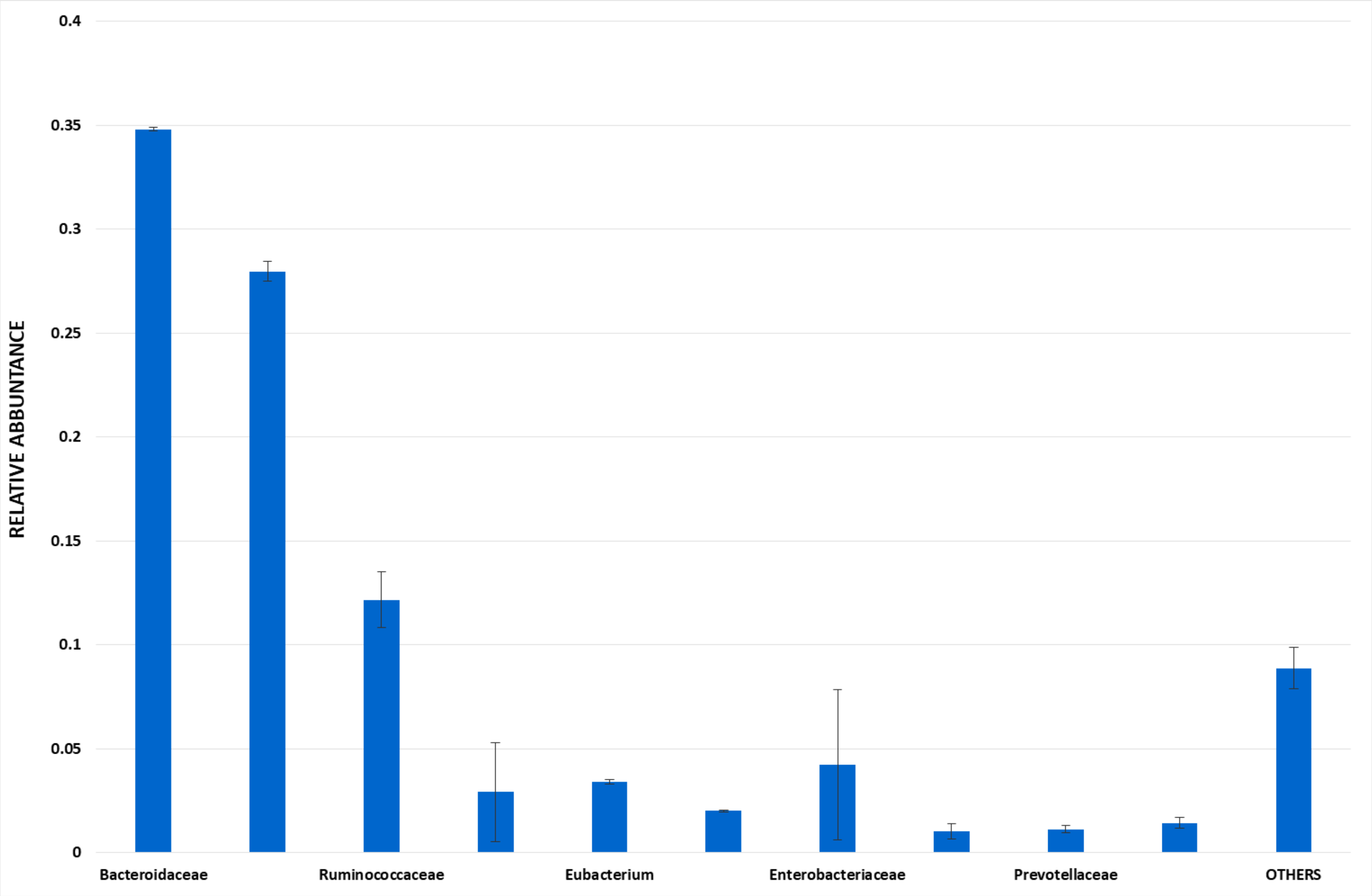

Figure S11. Relative abundance of families of the core bacterial microbiota of *S. cerevisiae* strain OS3.

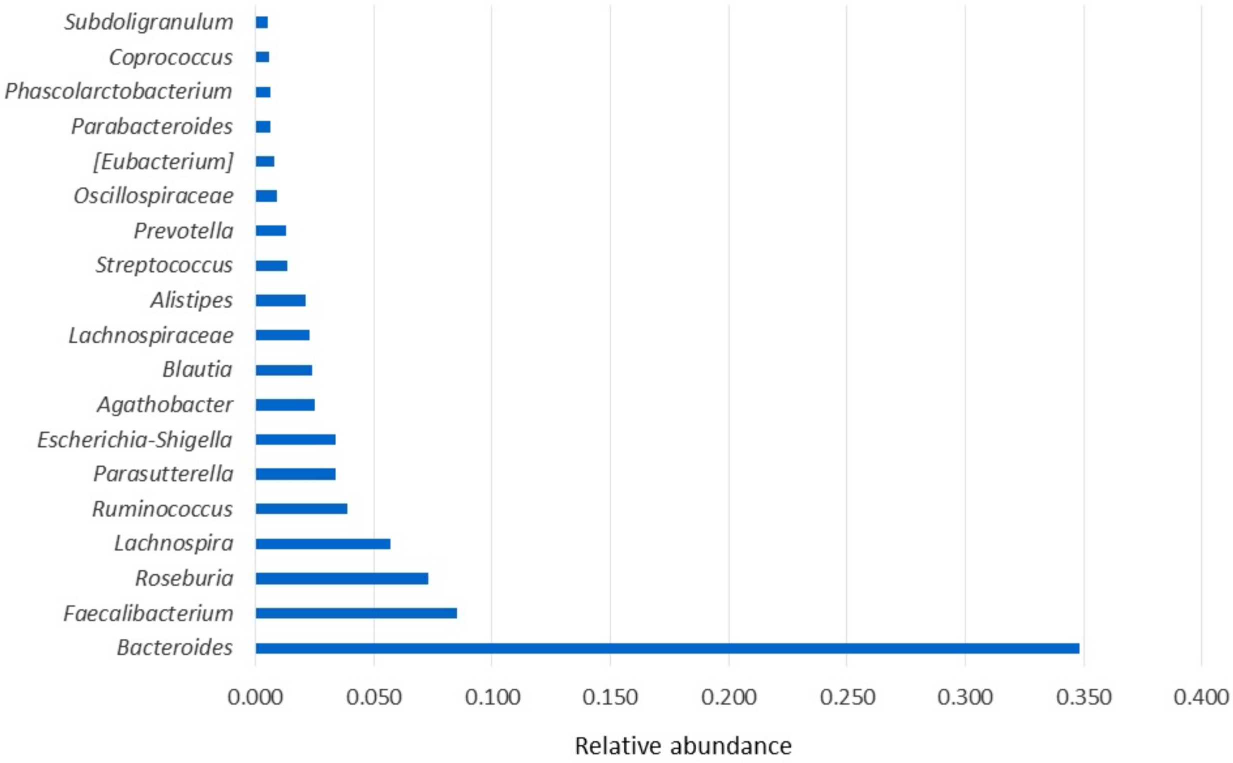

Figure S12. Relative abundance of genera of the core bacterial microbiota of *S. cerevisiae* strain OS3.

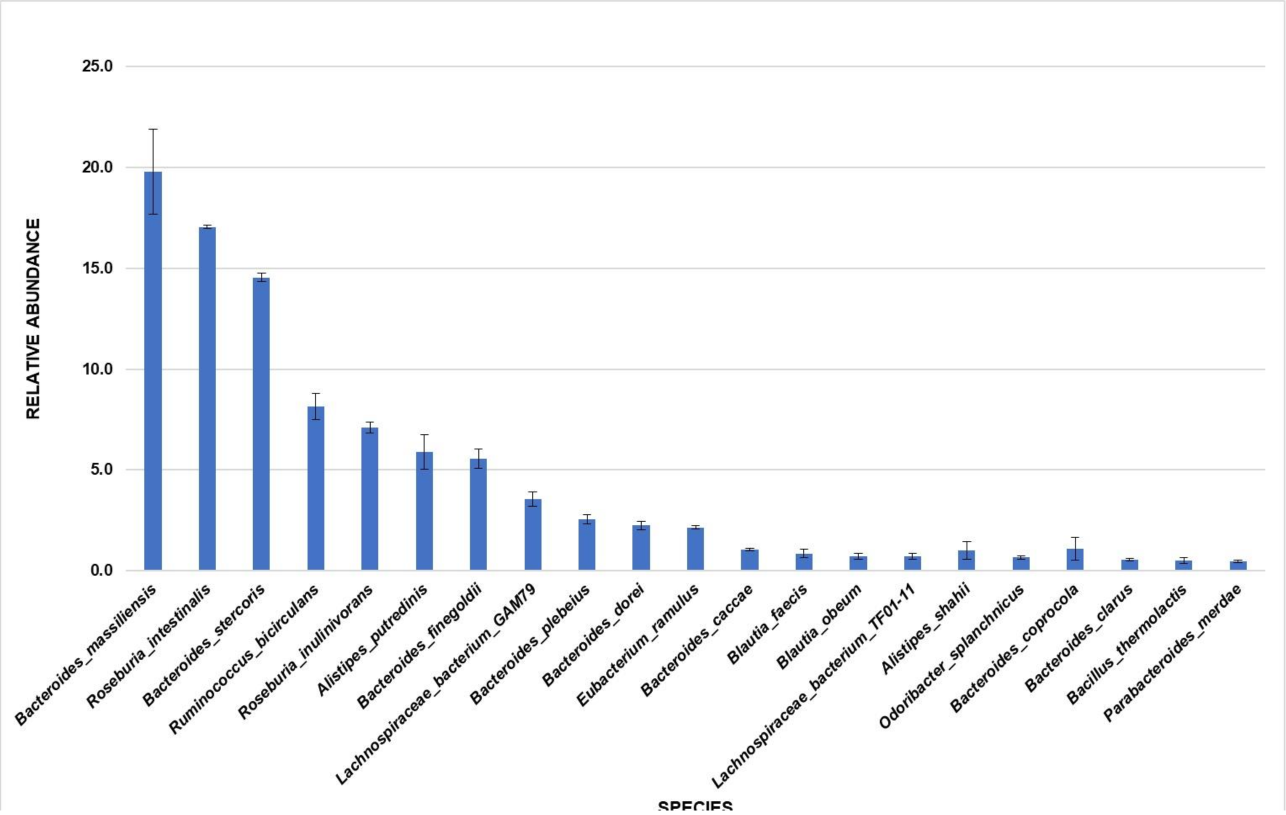

Figure S13. Relative abundance of species of the core bacterial microbiota of *S. cerevisiae* strain OS3.

Table S1: Protein concentration in crude enzymatic extracts of OS3 and V5 *S. cerevisiae* cells from different growth conditions.

Sample	Protein total (mg/ml)
S. cerevisiae OS3 at 24 h in YNBwAA	0.770 ± 0.070
S. cerevisiae OS3 at 200 h in YNBwAA	0.029 ±0.003
S. cerevisiae OS3 at 118 h in YSM+ 0.1g/L glu	0.059 ± 0.011
S. cerevisiae V5 at 24 h in YNBwAA	0.568 ± 0.023

Table S2: Multiple comparison of tyrosol production in extracellular medium of *S. cerevisiae* at different time-points of the fermentation process; padj values were obtained with one-way repeated measures ANOVA test and Bonferroni correction.

**Time-points comparison**	**Bonferroni p_{adj}**
6 h *vs.* 12 h	3.96×10^{-2}
6 h *vs.* 24 h	5.08×10^{-4}
6 h *vs.* 48 h	1.01×10^{-4}
6 h *vs.* 72 h	2.34×10^{-6}
12 h *vs.* 48 h	3.05×10^{-2}
12 h *vs.* 72 h	1.79×10^{-4}
24 h *vs.* 72 h	1.09×10^{-2}

Table S3. Qualitative evaluation of substrates hydrolysis by using glucanase activities in the crude extract of OS3 and V5 *S. cerevisiae* cells from different growth conditions after 48h of reaction.

**Substrate**	OS3- t24h in YNBwAA medium	OS3- t200h in YNBwAA medium	OS3- t118h in YSM medium	V5- t24h in YNBwAA medium
**Starch**	+/-	nd	nd	nd
**Amylose**	-	nd	nd	nd
**amylopectin**	+	++	+	+
**Pullulan**	+	++	-	-
**Glycogen**	-	+/-	+/-	-
**Curdlan**	-	nd	nd	nd
**β-Glucan**	-	nd	nd	nd
**Laminarin**	+	+	++	+++
**Laminaribiose**	+++	nd	nd	nd
**laminaripentaose**	+++	nd	nd	nd

−/+: percentage of products below 10%; +: 10–30% of products; ++: 30–60% of products; +++: percentage of products higher than 60%.; nd: the reaction was not carried out.

Video S1. Moving bacteria-like bodies (MBLBs) inside the cells of *S. cerevisiae* strain OS3 grown on YSM medium.
